# Construction of prediction models for growth traits of soybean cultivars based on phenotyping in diverse genotype and environment combinations

**DOI:** 10.1093/dnares/dsac024

**Published:** 2022-08-02

**Authors:** Andi Madihah Manggabarani, Takuyu Hashiguchi, Masatsugu Hashiguchi, Atsushi Hayashi, Masataka Kikuchi, Yusdar Mustamin, Masaru Bamba, Kunihiro Kodama, Takanari Tanabata, Sachiko Isobe, Hidenori Tanaka, Ryo Akashi, Akihiro Nakaya, Shusei Sato

**Affiliations:** Graduate School of Life Sciences, Tohoku University, Sendai, Miyagi 980-8577, Japan; Faculty of Agriculture, University of Miyazaki, Miyazaki 889-2192, Japan; Faculty of Agriculture, University of Miyazaki, Miyazaki 889-2192, Japan; Kazusa DNA Research Institute, Kisarazu, Chiba 292-0818, Japan; Graduate School of Medicine, Osaka University, Suita, Osaka 565-0871, Japan; Graduate School of Life Sciences, Tohoku University, Sendai, Miyagi 980-8577, Japan; Graduate School of Life Sciences, Tohoku University, Sendai, Miyagi 980-8577, Japan; Kazusa DNA Research Institute, Kisarazu, Chiba 292-0818, Japan; Kazusa DNA Research Institute, Kisarazu, Chiba 292-0818, Japan; Kazusa DNA Research Institute, Kisarazu, Chiba 292-0818, Japan; Faculty of Agriculture, University of Miyazaki, Miyazaki 889-2192, Japan; Faculty of Agriculture, University of Miyazaki, Miyazaki 889-2192, Japan; Graduate School of Medicine, Osaka University, Suita, Osaka 565-0871, Japan; Graduate School of Frontier Sciences, The University of Tokyo, Kashiwa, Chiba 277-0882, Japan; Graduate School of Life Sciences, Tohoku University, Sendai, Miyagi 980-8577, Japan

**Keywords:** soybean, field phenotyping, multiple sowing times, multiple regression models

## Abstract

As soybean cultivars are adapted to a relatively narrow range of latitude, the effects of climate changes are estimated to be severe. To address this issue, it is important to improve our understanding of the effects of climate change by applying the simulation model including both genetic and environmental factors with their interactions (G×E). To achieve this goal, we conducted the field experiments for soybean core collections using multiple sowing times in multi-latitudinal fields. Sowing time shifts altered the flowering time (FT) and growth phenotypes, and resulted in increasing the combinations of genotypes and environments. Genome-wide association studies for the obtained phenotypes revealed the effects of field and sowing time to the significance of detected alleles, indicating the presence of G×E. By using accumulated phenotypic and environmental data in 2018 and 2019, we constructed multiple regression models for FT and growth pattern. Applicability of the constructed models was evaluated by the field experiments in 2020 including a novel field, and high correlation between the predicted and measured values was observed, suggesting the robustness of the models. The models presented here would allow us to predict the phenotype of the core collections in a given environment.

## 1. Introduction

Soybean is one of the globally important crops that are rich in protein and lipid. Therefore, soybean has been used for food, oil, and industrial applications. As the demand has been highly increasing along with world population growth, soybean is cultivated all over the world. However, the cultivation area of each soybean cultivar is restricted to a relatively narrow range of latitude to achieve its higher yield based on the environment-dependent growth conditions, such as flowering time (FT) and vegetative growth.^1^^,^^2^ Therefore, the effect of climate changes is considered to be severe, and actually, the estimation that seed yield of soybean would be decreased to one-fourth by the 2080s relative to the 1980s has been reported.^3^ Considering these situations, controlling FT and vegetative growth, which are determined by multiple interactions between genes and environments,^4^ will be invaluable to maintain soybean yield under changing environmental conditions.

To address this issue, two major strategies have been undertaken to date; one is to develop cultivars that are tolerant to environmental changes based on genetic information (G) by applying classical breeding or genetic modification including genome-editing technology.^5^ A wide variety of cultivars or accessions has been established worldwide; 13,921 accessions have been collected throughout Japan, whereas 111,482 soybeans have been conserved in the world in 2020 according to Food and Agriculture Organization of the United Nations (http://www.fao.org/wiews). To enhance the studies on the accumulated soybean cultivars and assist in finding novel traits for crop improvement, mini core collections of Japanese soybean cultivars and world soybean cultivars have been established based on the genetic and agronomic traits characterizations.^6^

The other is to develop growth simulation models by measuring growth patterns in multiple environments (E). By applying environmental factors into an ideal simulation model, it outputs a predicted phenotype; such a simulation model allows us to make a planting plan in preparation for future changes in the environment. Many attempts have been made to construct growth simulation models of soybean using various approaches, such as simulations based on machine learning methods,^7^ and eco-physiological process.^8^^,^^9^ However, the number of genotypes or environments used to construct the mathematical models was limited. For instance, Nakano et al.^8^ simulated a soybean growth using only three genotypes, whereas Setiyono et al.^9^ designed a model only based on mid-latitudinal region in the USA and few cultivars mainly cultivated in the tested area. When the model constructed by Setiyono et al.^9^ was applied to different maturity groups of cultivars grown in different environments, low accuracy of prediction performance has been observed,^10^ indicating that a wide variety of tested genotypes as well as environmental conditions is crucial to accurately simulate growth patterns and provide the model with robustness. Furthermore, not only the G or E effect alone but also the interaction between G and E, termed G×E, results in phenotypic variations.^11–13^ Soybean growth and development are highly dependent on environmental factors, especially temperature change,^14^^,^^15^ while the effects vary greatly depending on the genotypic background.^16^ Significant effects of G×E on shoot architecture such as plant height, node number, and branch number have also been reported in soybeans.^17–19^

Since understanding the genetic basis for the G×E effect in growth dynamics is essential to simulate growth modelling, and solid genotype information is essential to accomplish this objective, we recently performed re-sequencing of representative soybean accessions in Japanese collections including Japanese landraces, cultivars, and world cultivars.^20^ Using high-density SNP markers obtained from the re-sequencing, we detected well-known loci for FT and seed colour-related traits by genome-wide association studies (GWAS).^20^ With the completion of whole-genome re-sequencing for the collections, our next purpose is to make a robust simulation model by accumulation of diverse phenotypes under varying environmental conditions.

In this study, we propose a new strategy to develop a growth simulation with the integration of G×E into the model. To achieve this goal, 93 soybean cultivars from the Japanese and world core collections were selected to increase the diversity of genotypes. These cultivars were grown in two fields in different latitude with multiple sowing times to increase the environmental variations. We evaluated the effects of the field and sowing time on flowering and growth phenotypes. GWAS were used to select a large set of genomic alleles associated with the flowering and growth, which could be input into the prediction model. We constructed the multiple regression models by fitting a parameterized model function to the prepared dataset and evaluated the constructed models by new dataset obtained from field experiments including a novel field.

## 2. Materials and methods

### 2.1. Plant materials, field experiment, and phenotyping

Field experiments were carried out in two experimental fields over 3 years starting from 2018. One was the field at University of Miyazaki (MF; 31.83°N, 131.41°E), located in the southern cultivated area of Japan, and the other was the field at Tohoku University (TF; 38.46°N, 141.09°E), located in the northern cultivated area of Japan. In 2020, we also used the experimental field at Kazusa DNA Research Institute (35.33°N, 139.99°E), located in the middle east cultivated area of Japan, for the purpose of evaluating the applicability of the model. In the fields at MF and Kazusa DNA Research Institute (KF), the climatic factors (average temperature, sunshine duration, and precipitation rate) were monitored by Automatic Weather Station Weather ROBO set in the field (Climatec, Inc.). In the field of TF, the climatic factors were provided by the Automated Meteorological Data Acquisition System (AMEDAS) at Kashimadai (38.46°N, 141.09°E), which is located in the field of TF.

Ninety-three soybean cultivars used in this study were listed in Supplementary Table S1, of which 84 consisted of Japanese core collection and 4 selected from world collections.^6^ A starter fertilizer containing N, P, and K, in the ratio 1.4:3.2:3.4 and 80 kg granular magnesium lime per 1,000 m^2^, were applied. The width of the row was designed as 80 cm with 40 cm intra-row spacing in each field, and seeding spot spacing was set as 60 cm. Before sowing, the seeds were kept in humid condition for about 4 days to reach ∼50% of moisture content, and three seeds were sown in a single spot and thinned into 1 plant after 2–3 weeks. In 2018, the seeds were sown on 24 July in MF and on 6 June in TF, and 8 and 3 individuals for each cultivar were planted in MF and TF, respectively. In 2019, at least two individuals were analysed in three sowing times, i.e. in June (12th in MF and 2nd in TF), July (23rd in MF and 2nd in TF), and August (16th in MF and July 30th in TF). In 2020, 6 individuals in MF and TF and 3 individuals in KF were analysed in three sowing times, in June (2nd in MF, 3rd in TF, and 9th in KF), July (7th in MF, 2nd in TF, and 9th in KF), and August (4th in MF, 30 July in TF, and 4th in KF). The total numbers of the individuals in the fields were summarized in Supplementary Table S2.

We investigated the flowering date at least once a week. We measured plant height (main stem length) once a week for all plants during a growing period and calculated the increment of plant height in a given week based on the difference from the previous week’s value. After the harvest, the terminal phenotype of plant height was measured. The trait values obtained by replication in each field were averaged for the analyses in this study. Distributions of the trait values of the individuals with replications in relation to each of the four sowing times in MF and TF were presented in Supplementary Fig. S1. The phenotype data used in this study were provided by the Supplementary data file.

### 2.2. Statistical analysis of field data

The relationship between FT and weekly growth of plant height as well as the relationship between FT and terminal plant height in individual fields was calculated based on Pearson’s correlation method in R software.^21^ To investigate the effect of genotype, environment, and G×E interaction on the terminal phenotypes, we performed an analysis of variance (ANOVA) based on the generalized linear model using gamma distribution to handle non-normally distributed data. ‘Cultivar’ indicated the variability of G effect whereas ‘environment’ indicated the variability of E effect (consisted of a combination of field × year × sowing time). For G×E, it was calculated as the interaction effect between cultivar (G) and environment (E). The ANOVA was carried out using ‘stats’ package from R (R Core Team^21^) and figures were produced using ‘ggplot2’ package.^22^

### 2.3. Parameterization of individual plant growth

By fitting a logistic function to the values of the plant height of an individual observed at intervals, we obtained its growth curve from the sowing. The logistic function describes the relation between the days from the sowing (*t*) and the plant height (*y*) and can be represented by an S-shaped curve that asymptotically approaches its upper bound (*K*) and lower bound (0), respectively, when *t* approaches positive and negative infinity (Supplementary Fig. S2A). In this study, we used the following definition to make the value of the function is 0 when *t* is 0:

$$y=\frac{\left(K+y_{0}\right)}{1+\left(\frac{K}{y_{0}}\right)e^{-rt}}-y_{0},$$

where *K* and *r* as well as *y*_0_ are the parameters that determine the shape of the curve (Supplementary Fig. S2B). We used *r* as an evaluation quantity related to the increasing rate of *y*. We empirically used 50 as a non-zero value for *y*_0_ for all the individuals and the maximum plant height of each individual for *K* to fit a logistic function to the observed data. Therefore, only the values of *r* were estimated by the fitting procedure. Note that fitting of the function was carried out, respectively, for each of the individuals. We carried out fitting of logistic function by using the ‘SciPy’ library available in the Python language.^23^

### 2.4. Multifactorial prediction models for growth-related traits

The values of *K* and *r* can determine the shape of the logistic function approximating the growth curve. Therefore, we constructed prediction models that estimate the values of *K* and *r* under given conditions. We used a linear model to characterize those quantitative traits. The linear model accepts genetic factors (G), environmental factors (E), and their interactions (G×E) as the explanatory variables and calculates the trait value. The values of the explanatory variables provided as the inputs are multiplied with their coefficients and then summed up to obtain an estimation of the trait. When we have individuals that are indexed from 1 to *N*, the observed value of the i-th individual is expressed as follows:

$$y_{i}=c+\sum_{h=1}^{m}\alpha_{h}x_{h,i}+\sum_{k=1}^{n}\beta_{k}z_{k,i}+\sum_{h=1}^{m}\sum_{k=1}^{n}\gamma_{h,k}x_{h,i}z_{k,i}+\varepsilon_{i,}$$

where h and k are index variables and m and n are, respectively, numbers of the explanatory variables corresponding to the genetic and environmental factors. $x_{h,i}$ and $z_{k,i}$ numerically encode the h-th genetic factor (G) and the k-th environmental factor (E) of the i-th individual. $\alpha_{h}$ and $\beta_{k}$ are the coefficients of those factors. Here, $x_{h,i}$ is a binary dummy variable that corresponds to a genetic factor and indicates the absence or presence of a specific sequence variant by whether its value is 0 or 1. $z_{k,i}$ is a numerical variable that represents the value of an environmental factor such as average temperature, sowing time, and latitude of field. $x_{h,i}z_{k,i}$ is the interaction between the h-th genetic factor and the k-th environmental factor (G×E). $\gamma_{h,k}$ is the coefficient of the interaction between $x_{h,i}$ and $z_{k,i}$. Since the existence of a genetic factor is encoded by a binary variable $x_{h,i}$, the value of $x_{h,i}z_{k,i}$ is 0 or $z_{k,i}$ depending on the value of $x_{h,i}$, representing the selective effect of the environmental factor in a natural manner. c is a constant. $\varepsilon_{i}$ is an error term. Here, $x_{h,i}$ and $z_{k,i}$ are observed values whereas $\alpha_{h}$, $\beta_{k}$, $\gamma_{h,k}$, and c are model parameters to be determined. Those model parameters are determined so that the model values calculated by the explanatory variables are concordant with the trait values observed.

### 2.5. Selection of factors for prediction models

We prepared candidates of the genetic factors (G) using variants of DNA sequences obtained by the whole-genome sequencing (WGS) in the previous work.^20^ The WGS data based on the reference genome Gmax_275_v2.0 softmasked sequences consisted of 10,116,707 SNPs and 2,835,680 indels (at 12,952,387 variant positions in total) annotated as ‘PASS’ by the GATK variant caller.^24^^,^^25^ Among those positions, we selected 207,944 variant positions whose putative impact annotated by the SnpEff software^26^ were ‘HIGH’ or ‘MODERATE’. The categorization of the selected variants in the 93 cultivars in relation to whether they were SNPs or indels, bi-allelic loci with a single variant allele or multi-allelic loci with multiple variant alleles, and homozygous or heterozygous is presented in Supplementary Fig. S3. We used the bi-allelic loci (156,362 positions in total) in the GWAS for the traits.

We obtained the *P*-values for the GWAS by using the Hail library (version 0.2.93) available in the Python language.^27^ To take the genetic relatedness and population structure latent in the dataset into account, we first generated a relationship matrix of the 93 cultivars by the ‘realized_relationship_matrix’ function using all the genotypes at the selected bi-allelic loci (Supplementary Fig. S4A). This matrix presented the pairwise measures of genetic relatedness among the cultivars. We confirmed the relationship among the cultivars that the matrix presented by the result of hierarchical clustering based on the genetic relatedness (Supplementary Fig. S4B).

We then prepared a linear mixed model (null model) including the fixed effect consisting of only an intercept and the random effects reflecting the genetic background. We described the model by the ‘linear_mixed_model’ function using the relationship matrix as input. We determined the model parameters including the variances of the residuals by the ‘fit’ function. We also prepared a linear mixed model (alternative model) by adding the variant effects at the testing locus to the fixed effects of the null model and determined the model parameters. We calculated the *P*-value at the testing locus by a likelihood ratio test of the two models using a chi-squared test. We iterated the calculation of the *P*-values for the loci along the chromosomes by the ‘linear_mixed_regression_rows’ function. We plotted the *P*-values in the scale of the negative of the base 10 logarithm [-log_10_(*P*-value)] along the chromosomes as the Manhattan plots.

We iterated the GWAS procedure for the eight environments (i.e. four different sowing times in 2018 and 2019 in two fields TF and MF). To select the candidates of the genetic factors for the model of a trait, we introduced a heuristic method as follows. In each environment, we first sorted the chromosomal positions used in the GWAS in the increasing order of their *P*-value. From the sorting result, we obtained the top *N* significant chromosomal positions (we used *N *=* *100 in this study). We then accumulated the top *N* significant chromosomal positions from all the eight environments and generated the initial set of the candidates of the genetic factors by excluding the duplication. This initial set contained the chromosomal positions that were commonly significant in most of the environments as well as the ones that were significant in one or a few environments. The latter could play important roles in the interaction between the genetic factors and the environmental factors (G×E) and used in the following models as G×E explanatory variables.

The iteration of the GWAS procedure for the eight environments generated eight *P*-values for each chromosomal position. In relation to a trait, we defined the statistical significance of a chromosomal position by the minimum value of the eight *P*-values. We then sorted the chromosomal positions in the initial set of the candidates of the genetic factors prepared as above according to their statistical significance and put them into the priority list of the candidates of the genetic factors one by one. If the minimum value of the distances of a chromosomal position to the members already in the priority list was less than a predefined threshold of 100 kb, such the chromosomal position was discarded and not included into the priority list. The priority lists indicating the orders of the chromosomal positions for traits FT, *K*, and *r* are presented by Supplementary Tables S3–S5. In the tables, associated *P*-values and adjusted *P*-values (*q*-values) by the Benjamini–Hochberg procedure for estimation of the false discovery rate (FDR)^28^ are also presented.

The chromosomal positions were converted to explanatory variables each of which shows whether an individual has a specific genotype (i.e. a pair of allele types) by using numerical values 1 or 0. We evaluated the correlation between a pair of explanatory variables by the Pearson’s correlation coefficient between their profiles consisting of the values in all the cultivars in the eight environments. Prepared candidates of explanatory variables in the priority list were added to the model in an incremental manner up to the indicated number based on the significance level in GWAS under the condition that the absolute values of the correlation coefficient among all the explanatory variables remained less than a predefined threshold of 0.5. In the procedure of model construction, we utilized only the homozygous genotypes and excluded the heterozygous genotypes under the assumption that the heterozygous ones were rare in the soybean germplasms because they were genetically fixed (Supplementary Fig. S3). Furthermore, those heterozygous genotypes could be different from the genotyped lines^20^ in some environments and hence were not suitable for model construction. We excluded the candidates of the explanatory variables when the number of the cultivars that had the genotype was less than two or the number of the cultivars that did not have the genotype was less than two.

We prepared average temperatures of 10 periods after the sowing (indicated by the variables T_0_, T_10_, …, T_90_) as the candidates of environmental factors (E) and added them to the model in chronological order. We included a candidate period into the model if the absolute values of the correlation coefficient between the candidate and all the periods already in the model were less than a predefined threshold of 0.5. The correlations among the candidate periods could be determined by the training data (Supplementary Fig. S5). If we select T_0_ as the first choice, only T_30_ could satisfy the condition above. Therefore, we used T_0_ and T_30_ for all the models in this study. We also used the sowing time (in the days from the beginning of the year) and the latitude of the field to include the effects of the day length as the environmental factors in the model.

We then defined the interactions between genetic factors and environmental factors (G×E) by all the pairs of the explanatory variables so that the selective effects of a sequence variant (G) to either of the average temperatures, the sowing time, and the latitude (E) could be reflected in the model. The value of an interaction term can be computed by the product between a binary variable representing a binary encoded sequence variant and a numerical variable representing either of the average temperatures, the sowing time, or the latitude. The binary variable, known as a dummy variable, indicates the absence or presence of a specific sequence variant by whether its value is 0 or 1. If the sequence variant is present, the value of the binary variable is 1 and its product with the value of the counterpart of the interaction remains non-zero and can contribute to the model value, representing the selective effect of the sequence variant to the environment.

### 2.6. Determination of model parameters

We determined the values of the parameters in the models by using the ordinary least squares function of the ‘SciPy’ library available in the Python language. We used the observed values of the 93 cultivars in the eight environments comprising of four different sowing times in 2018 and 2019 in two fields (TF and MF) as the training data and then evaluated the obtained models using the observed values of the 32 cultivars in the nine environments comprising of three different sowing times in 2020 in the three fields (TF, KF, and MF) as the test data. We used the average of the trait values when there were replications of observations on a cultivar and multiple trait values were available. We can symbolically denote the models using the trait (P), genetic factors (G), environmental factors (E), and their interactions (G×E). Depending on whether the factors G, E, and G×E are used in the model or not, there can be several patterns of the models represented as P = G (only the genetic factors), P = E (only the environmental factors), P = G + E (both of the genetic and environmental factors), and P = G + E + G×E (all of the genetic and environmental factors and their interactions). We examined the prediction ability of the models adopting the patterns above by increasing the number of the explanatory variables corresponding to the genetic factors from 1 to 100. We used the Pearson’s correlation coefficient (*R*) and the root mean squared error (RMSE) for evaluation of the concordance between the observed values and the model values for estimation and prediction, respectively, in the training and test data.

## 3. Results

### 3.1. Effect of field and sowing time on phenotypic variation among soybean cultivars

A total of 93 soybean cultivars with a wide variety of genotypes, mainly selected from the Japanese core collection, were used in this study (Supplementary Table S1). These soybean cultivars were grown in two fields, the experimental fields at MF, located in the southern cultivated area of Japan, and the experimental field at TF, located in the northern cultivated area of Japan. The environmental conditions of these fields in the years of field experiments (2018 and 2019) were summarized in Supplementary Fig. S6. In 2018, we evaluated the phenotypic data by applying standard cultivation period of each area, i.e. sowing in July at MF and sowing in June at TF. In 2019, on the other hand, multiple sowing time strategies, i.e. sowing in June, July, and August in each field, were applied with the aim to increase environmental variations. For the target phenotypes, flowering status and plant height (main stem length) were measured once a week to obtain the basic information for growth modelling. In addition, terminal phenotype of the plant height was measured after harvesting.

**Figure 1:**
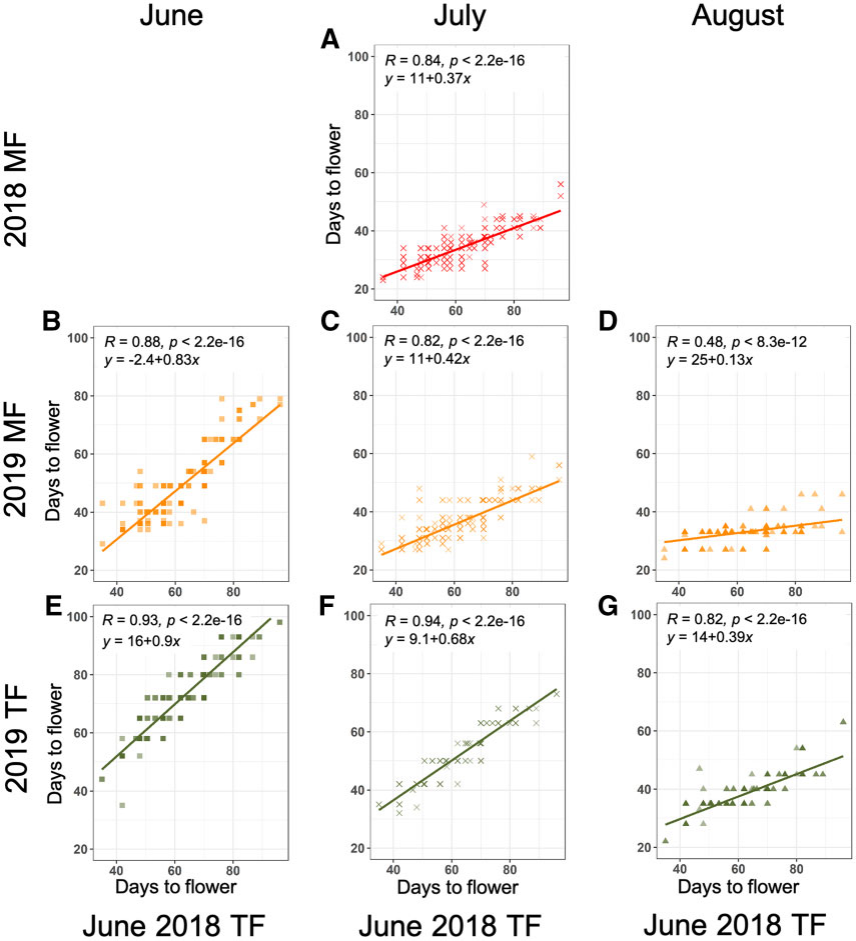
Scatter plots depicting the FTs for all 93 soybean cultivars in two fields and across three different sowing times compared with the FT in June 2018 in TF. (A) July 2018 in MF; (B) June 2019 in MF; (C) July 2019 in MF; (D) August 2019 in MF; (E) June 2019 in TF; (F) July 2019 in TF; (G) August 2019 in TF. The correlation and *P-*value were calculated based on Pearson’s correlation.

To evaluate the effects of field and sowing time, FT observed in each field and sowing time was compared using the dataset obtained in June 2018 in TF as a reference (Fig. 1). Regarding sowing in July in MF, FT patterns were similar between 2018 and 2019 (*R *=* *0.84 and 0.82, regression coefficients = 0.37 and 0.42), which became ∼20 days earlier in early-flowering cultivars and 40 days earlier in late-flowering cultivars compared with the reference (Fig. 1A and C). In the case of sowing in June, FT became about 10 days earlier in early-flowering cultivars to 20 days earlier in late-flowering cultivars in MF (*R *=* *0.88, regression coefficient = 0.83; Fig. 1B). In TF, the FT in June 2019 was delayed 10 days in all cultivars compared with that in June 2018 (*R *=* *0.93, regression coefficient = 0.90; Fig. 1E), presumably due to lower temperature in June 2019 (Supplementary Fig. S6A). In July-sowing in TF, the FT became about 5 days earlier in early-flowering cultivars to 20 days earlier in late-flowering cultivars (*R *=* *0.94, regression coefficient = 0.68; Fig. 1F). When sown in August, almost all cultivars flowered from 30 to 40 days in MF and the FT showed ∼10 days earlier in early-flowering cultivars to 50 days earlier in late-flowering cultivars compared with that in June 2018 in TF (*R *=* *0.48, regression coefficient = 0.13; Fig. 1D). In TF, early-flowering cultivars flowered as is the case in August 2019 in MF, while late-flowering cultivars flowered 10 days later than that in MF (*R *=* *0.82, regression coefficient = 0.39; Fig. 1G). We also compared the FTs among eight environments by making the correlation matrix (Supplementary Fig. S7) and found that all combinations of the conditions showed positive correlation. In terms of cultivar comparison, the order of FT was conserved in two fields and three sowing times.

To evaluate the effect of sowing time on the growth phenotype, correlation analysis between FT and weekly growth of plant height was carried out (Supplementary Figs S8 and S9). At the fourth week after sowing, FT was negatively correlated with the growth rate in both fields and all sowing time (*R *=* *−0.31 to −0.07; Supplementary Figs S8A, E, I, M and S9A, E, I, M), indicating that early-flowering cultivars tended to grow faster in the early growth phase. At the fifth week in MF, correlation became positive in cultivars sown in July and August (*R *=* *0.17 to 0.46; Supplementary Fig. S8F, J, and N), while it remained flat in cultivars sown in June (*R *=* *0.07; Supplementary Fig. S8B). At the sixth week in MF, correlation became positive with high growth rate in both early and late flowering in June sowing (*R *=* *0.31; Supplementary Fig. S8C), while the diminished growth rate was observed in early-flowering cultivars sown in July and August 2019 except ones with indeterminate genotypes (Supplementary Fig. S8K and O). At the seventh week in MF, the diminished growth rate was observed in both early- and late-flowering cultivars sown in June and July (Supplementary Fig. S8D, H, and L). In TF, negative correlation was observed in all three sowing times at the fourth week (*R *=* *−0.27 to −0.07), especially the high growth rate in early-flowering cultivars enhanced the negative correlation in August (*R *=* *−0.27; Supplementary Fig. S9M). Correlation turned positive with the high growth rate of late-flowering cultivars sown in August at the sixth week (*R *=* *0.45; Supplementary Fig. S9N), while correlation remained flat and negative in June (*R *=* *−0.09 and 0.03) and July (*R *=* *−0.25; Supplementary Fig. S9B, F, and J). Correlation turned positive in all of three sowing times in TF at the eighth week (*R *=* *0.02 to 0.63), and the diminished growth rate of early-flowering cultivars was observed in July and August (Supplementary Fig. S9C, G, K, and O). At the 10th week in TF, the high growth rate was observed in late-flowering cultivars sown in June 2019 (*R *=* *0.36; Supplementary Fig. S9H), while diminished growth expanded to late-flowering cultivars in both June 2018 and July (Supplementary Fig. S9D and L). These results suggest that the period of the high growth rate was significantly affected by sowing time as well as fields. Thus sowing time shifts resulted in increasing phenotypic variations under different environmental conditions.

**Figure 2:**
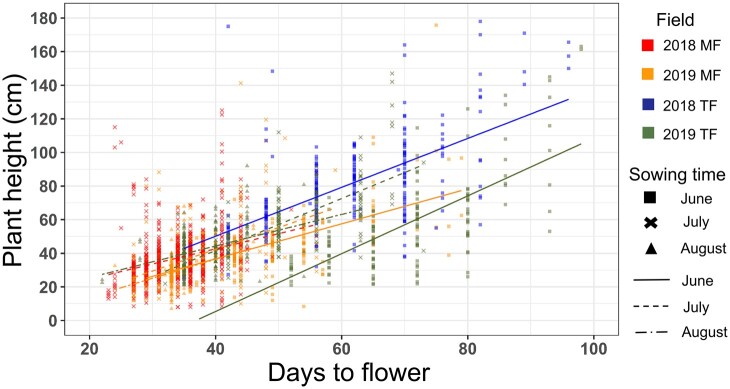
Relationship between FT and terminal plant height. Red and orange represent MF in 2018 and 2019. Blue and green represent TF in 2018 and 2019. Square, cross, and triangle dots represent individual cultivar sown in June, July, and August, respectively. Solid, dashed, and dotted-dashed lines represent regression lines for cultivars sown in June, July, and August, respectively (A color version of this figure appears in the online version of this article).

Regarding the effects of sowing time against the terminal phenotype of plant height, the ANOVA results strongly suggest that plant height was significantly affected by the interaction between cultivar and sowing time (G×E; Table 1). To evaluate the relationship between the FT and terminal phenotype of plant height in three sowing times and two fields, the FT and each terminal phenotype were plotted (Fig. 2 and Supplementary Table S2). As shown in Fig. 2, correlations between the FT and plant height were observed in each field and sowing time. In June 2019 TF, the FT was delayed in comparison to June 2018 TF as described above (Fig. 1E), and in addition, the range of plant height shifted to become shorter. The correlation between FT and plant height could be conserved between June 2018 TF and June 2019 TF populations. Significantly lower temperature, ∼3–5°C on average (Supplementary Fig. S6), during late-June to mid-July in 2019 compared with 2018 could be the cause of delay in flowering and shorter length in main stem in June 2019 TF. Thus, the temperature in the initial phase of growth greatly influenced growth, suggesting the temperature effect should be considered to construct a prediction model.

**Table 1:** ANOVA for variation of plant height phenotype among soybean cultivars grown in 2018 and in three different sowing times in 2019 in MF and TF

	df	Sum Sq	Mean Sq	*F*-value	Pr(>*F*)
2018 MF and TF					
Cultivar (G)	92	224,731	2,443	18.486	<2e−16^***^
Field (E)	1	354,101	354,101	2,679.742	<2e−16^***^
G × E	91	69,172	760	5.753	<2e−16^***^
Residuals	745	98,444	132		
2019 MF					
Cultivar (G)	92	97,857	1,064	10.298	<2e−16^***^
Sowing time (E)	2	27,691	13,845	134.047	<2e−16^***^
G × E	183	31,592	173	1.671	9.43E−06^***^
Residuals	443	45,756	103		
2019 TF					
Cultivar (G)	92	130,939	1,423	12.135	<2e−16^***^
Sowing time (E)	2	30,117	15,059	128.395	<2e−16^***^
G × E	175	64,215	367	3.129	1.90E−15^***^
Residuals	215	25,216	117		
All data					
Cultivar (G)	92	389,813	4,237	35.089	<2e−16^***^
Field × year × sowing time (E)	7	506,342	72,335	599.03	<2e−16^***^
G × E	633	220,702	349	2.887	<2e−16^***^
Residuals	1,403	169,416	121		

*** Significant differences at the level of *P *<* *0.0001.

### 3.2. GWAS of phenotypes in different fields and sowing times

As we obtained phenotypic data under different fields and sowing times, we conducted GWAS for FT and plant height against 156,362 SNPs and indels genotyped based on our previous re-sequencing data.^20^ Results of GWAS for FT were shown in Supplementary Figs S10, S11 and Table S6. We used 1% and 5% FDR (*q *<* *0.01 and *q *<* *0.05) as the thresholds for statistical significance. As expected, SNPs associated with two major FT loci, *E2* (chr10_45310798)^29^ and *E3* (chr19_47623288),^30^ were identified in both fields. However, the significance of these alleles was different between the two fields. In TF, *E2* and *E3* were detected as top peaks of the Manhattan plots in both June 2018 and 2019, while the SNP on *Glyma.12G073900* (chr12_5520945) encoding two-component response regulator related to FT was detected as the top peak in June and July 2019 in MF. In the context of field-specific associations, the SNP on *Glyma.07G166200* (chr07_25705280) was identified in June 2019 in MF, while in TF the SNP on *Glyma.08G013500* (chr08_1057296) was identified in June 2019. The early-flowering tendency in MF compared with TF might be the cause of these variations between two fields. Similarity of GWAS results between August 2019 in TF with an early-flowering trend and June 2019 in MF, in which *Glyma.12G073900* was detected as top peak followed by *E2* would support this estimation.

For plant height, the SNPs associated with *Dt1/GmTFL1* (chr19_45208498) for determinate stem growth habit^31^ were identified in all conditions (Supplementary Figs S12, S13 and Table S6). In addition, the SNP on *Glyma.12G076000* (chr12_5829324) was detected in August 2019 MF and July 2019 TF, and a cluster of SNPs and indels is in the genome region of chr9_3247955-chr9_3273449 correspond to *Glyma.09G038700* and *Glyma.09G039000* were identified in June 2019 MF, July 2018 MF, August 2019 MF, and June 2018 TF. These results indicated overall similarity in the genotype–phenotype relation among the population used under tested environments. This feature would be an advantage to create the growth model based on the plant height as target phenotype.

### 3.3. Prediction models of growth-related traits interacting with the environment

By applying multiple sowing times in 2019, we could accumulate phenotypic data with a variety of genotype and environmental combinations. Thus, we conducted the construction of multiple regression models using phenotypic data obtained in 2018 and 2019. We constructed the prediction models of FT, *K* (maximum value of plant height), and *r* (growth rate) as the growth-related traits (details were described in Sections 2.3–2.6, and results of GWAS in *r* are shown in Supplementary Figs S14 and S15). As already described earlier, we set the threshold of the absolute value of the correlation coefficient to 0.5 while we added the explanatory variables corresponding to the sequence variants and the temperatures to the models in an incremental manner. Under this condition, we could select T_0_ and T_30_ from the candidates, respectively, corresponding to the average temperatures in the periods from the sowing and a month later. Hereafter, we commonly used T_0_ and T_30_ in addition to the sowing time and the latitude of the field in the environmental factors of the models. The sequence variants as the genetic factors in the models were selected so that they were located at least 100 kb apart from each other. Changing the number of genetic factors from 1 to 100, we constructed models using four patterns (P = G, P = E, P = G + E, and P = G + E + G×E), and then evaluated the relationship between the complexity of the models and their prediction ability (Supplementary Figs S16–S18). The details of the 100 genetic factors used for traits FT, *K*, and *r* are, respectively, presented by Supplementary Tables S7–S9 and S10–S12.

**Figure 3:**
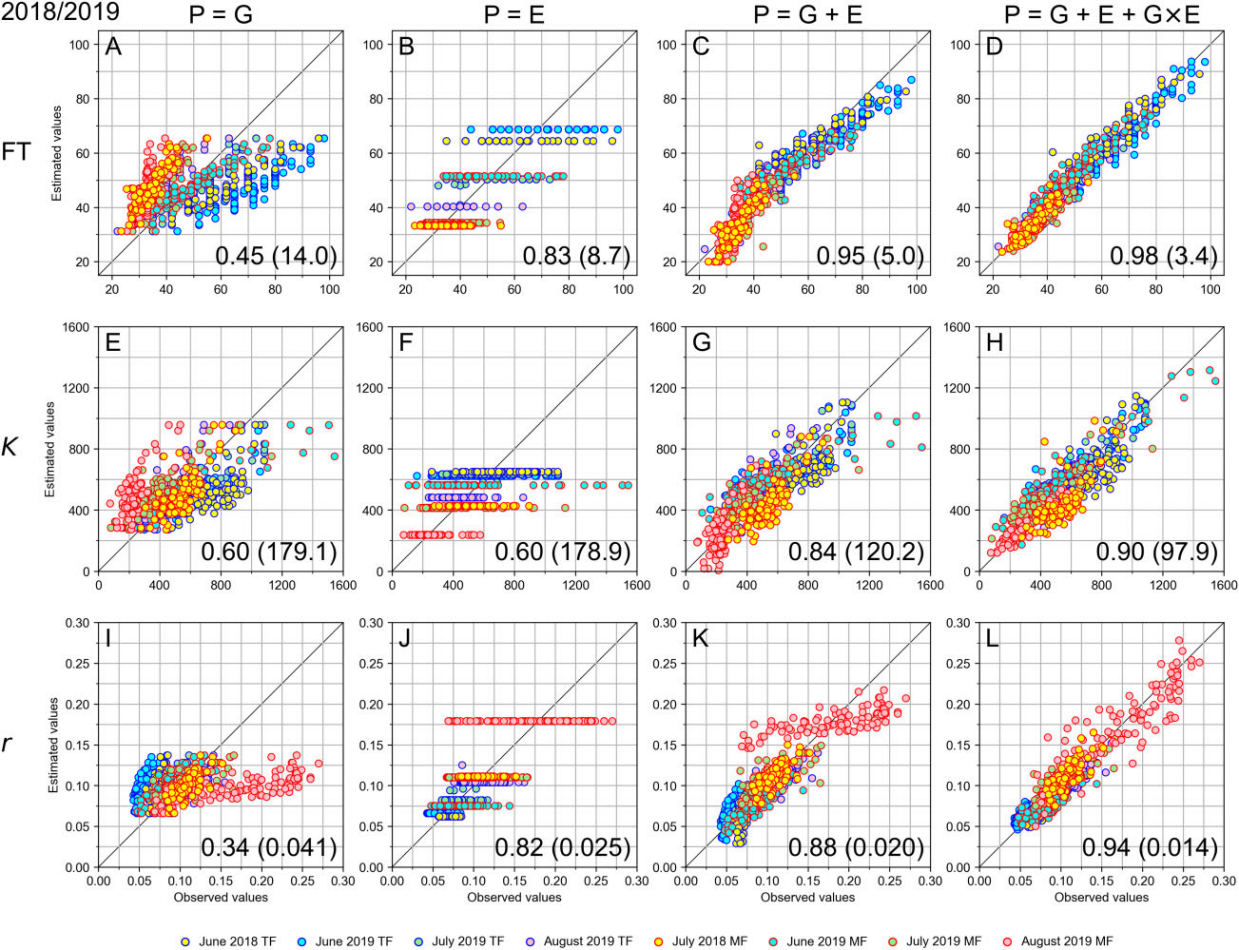
Scatter plots depicting the relationships between the observed values and the estimated values of the traits FT, *K*, and *r* calculated by the models with 50 genetic factors using the training data in 2018 and 2019. (A–D) The estimated values of FT were, respectively, calculated by the models P = G, P = E, P = G + E, and P = G + E + G×E; (E–H and I–L) the estimated values of *K* and *r* were calculated in the same way; the horizontal axis and the vertical axis show the observed values and the estimated values, respectively. Samples in TF and MF are indicated by small circles with blue and red edge colour. Samples observed in June, July, and August in 2019 are coloured in cyan, light green, and light red. Samples in 2018 are coloured in yellow. The Pearson’s correlation coefficient between the observed values and the estimated values is indicated at the bottom right of each plot. The RMSE is also indicated in the parentheses (A color version of this figure appears in the online version of this article).

Figure 3 shows the scatter plots of the relationship among the estimated values by the model using 50 genetic factors and the observed values in the training data. With the models consisting only of the genetic factors (P = G), the samples of an identical cultivar had the identical values in any environment and could not reflect the environmental conditions. The eight datasets constituting the training data in eight colours had the same distributions of the estimated values in the vertical direction in the scatter plots (Fig. 3A, E, and I). With the models using only the environmental factors (P = E), the samples in the identical environments had the identical values that were determined by the average temperatures (T_0_ and T_30_), the sowing time, and the latitude of the field. The estimated values of the eight datasets were aligned in the horizontal direction in the scatter plots (Fig. 3B, F and J). On the other hand, the models using the environmental factors in addition to the genetic factors (P = G + E) could include the additive effects by the average temperatures, the sowing time, and the latitude of the field as well as the 50 genetic factors (Fig. 3C, G, and K). Since the effects by the environmental factors differed in the eight environments, the ranges of the estimated values could be distributed in the vertical directions. However, since the magnitude relationship among the estimated values in each of the environments was determined solely by the identical genetic factors, all the environments had the identical variance of the distribution of the estimated values and only the base values determined by the environmental factors differed with each other, and therefore looked as if they were generated by translating the identical distribution of the values in the vertical direction. With the models consisting of the genetic and environmental factors and their interactions (P = G + E + G×E), the offset values according to the environments were added to the estimated values even for individuals of an identical cultivar, reflecting the environmental condition to the distributions of the estimated values (Fig. 3D, H, and L). The assigned parameters for each of G, E, and G×E in the models (P = G, P = E, P = G + E, and P = G + E + G×E) are presented by Supplementary Tables S13–S24.

### 3.4. Genetic factors selected for the model construction

The genetic factors selected for the model construction were summarized in Supplementary Tables S7–S9 and S10–S12. Regarding the genetic factors selected for the FT model, alleles of major flowering, *E2* and *E3*, were selected in top three genetic factors (FT02 and FT03), while allele of determinate growth, *Dt1*/*GmTFL1*, was selected on FT11. In addition, an allele of the SNP on *Glyma.12G073900*, which was identified in GWAS on FT in June 2019 MF, July 2018 MF, July 2019 MF, June 2018 TF, July 2019 TF, and August 2019 TF was selected as FT01. Alleles of the SNPs on the genes encoding TIR-NBS-LRR class disease resistance proteins and other disease resistance proteins, *Glyma.11G022700* (FT21), *Glyma.19G134200* (FT33), *Glyma.16G215300* (FT57), *Glyma.06G259800* (FT63), *Glyma.19G134100* (FT64), *Glyma.03G087700* (FT84), and *Glyma.13G187900* (FT86), were selected for the FT model. The SNPs identified in GWAS on FT in MF or TF were also selected in the FT model, such as chr08_1057296 on *Glyma.08G013500* encoding serine ammonia-lyase (FT04) and chr16_20809600 on *Glyma.16G102800* encoding zinc ion binding protein (FT05).

For the genetic factors selected for the *K* model, alleles associated with *Dt1/GmTFL1* were selected as K01, while alleles associated with *E2* and *E3* were selected as K09 and K03, indicating the high contribution of both determinate growth habit and FT in the model for *K*. In addition, an allele of the SNP, chr04_44462957, which was identified in GWAS on PH in August 19 MF, on *Glyma.04G179400* encoding thiol-disulphide oxidoreductase was selected as K08, and an allele of the SNP, chr05_35695296, which was identified in GWAS on PH in June 19 TF, on *Glyma.03G140800* encoding AP2 domain transcription factor was selected as K32. In the *K* model, four variant sites on the gene related to plant hormone signalling were selected, i.e. chr09_40442457 on *Glyma.09G179600* encoding aminopeptidase M1 (K16), chr20_41782778 on *Glyma.20G180000* encoding auxin response factor (K40), chr19_46732225 on *Glyma.19G213900* encoding drought-responsive family protein (K56), and chr10_44287403 on *Glyma.10G210600* encoding auxin response factor (K96). As is the case in the model of FT, *K* model contains number of alleles on the genes related to defense response, such as chr08_15511073 on *Glyma.08G192900* encoding TIR-NBS-LRR disease resistance protein (K45), chr15_11651285 on *Glyma.15G142400* encoding glucan endo-1,3-beta-glucosidase (K60), chr07_5726747 on *Glyma.07G064100* encoding NB-ARC domain-containing disease resistance protein (K79) and chr02_45790034 on *Glyma.02G274800* encoding leucine-rich repeat receptor-like protein kinase family protein (K99).

For the genetic factors selected for the *r* model, *E2*, *E3*, and *Dt1/GmTFL1* were selected (r03, r04, and r09, respectively) from alleles of major flowering and determinate growth loci, indicating both flowering and determinate growth habit traits affect the growth rate. Another FT-related allele, chr12_5520945 on *Glyma.12G073900* encoding two-component response regulator was selected as r14. The indel variant selected as r65 was chr11_5982489 on *Glyma.11G079500* encoding a homolog of *Arabidopsis MCA1* that involved in mechano-stimulated calcium uptake mechanism and in mechanosensing in the primary root. SNPs on the other gene related to root development were also selected in the *r* model, i.e. chr16_31864920 on *Glyma.16G158400* encoding multicopper oxidase (r12). Similar to FT and *K* model, *r* model contained alleles on defense-related genes, i.e. chr07_5719283 on *Glyma.07G064100* encoding NB-ARC domain-containing disease resistance protein (r13), chr18_7973445 on *Glyma.18G082100* encoding RPM1-like disease resistance protein (r19), chr16_33803483 on *Glyma.16G176700* encoding WRKY family transcription factor (r53), chr06_1291246 on *Glyma.06G017500* encoding PBL21-like serine/threonine protein kinase (r62), and chr19_39524087 on *Glyma.19G134200* encoding putative disease resistance protein (r84).

### 3.5. Evaluation of the prediction models

To provide the phenotype data for evaluation of the created models, we selected 32 cultivars from 93 tested ones and cultivated with three sowing times in MF and TF in 2020. In addition, to test the applicability of the created models to the different field conditions, we cultivated the same 32 cultivars in the experimental field of Kazusa DNA research Institute (KF) located in Kanto, a middle east cultivated area of Japan (A3 region^5^). Environmental conditions in three fields were summarized in Supplementary Fig. S19, and FT observed in 2020 were shown in Supplementary Fig. S20. By applying the accumulated environmental and phenotypic data, the constructed models were evaluated. We first evaluated the relationship between the number of the genetic factors in the models and the prediction ability using the samples in TF, KF, and MF observed in 2020 (Supplementary Fig. S21). When the number of the genetic factors in the models increased from 1 to 100, the Pearson’s correlation coefficient indicating the concordance between the observed and predicted values in the samples in 2020 of FT, *K*, and *r*, respectively, achieved the maximum values 0.93, 0.90, and 0.58. The correlation coefficient increased with increasing the number of the genetic factors and reached to the plateaus. For the succeeding analysis, therefore, the models using 50 genetic factors harbouring the relevant genes were adopted as sufficient from the viewpoint of the prediction ability. The scatter plots showing the improvement process of the concordance between the observed values and the estimated values by the models P = G, P = E, P = G + E, and P = G + E + G×E are presented in Supplementary Figs S16–S18. The distribution of the genetic factors in the 93 cultivars is presented in Supplementary Fig. S22, showing the bidirectional relationships of the variant patterns among the cultivars as well as among the genetic factors. Relationships between the genotypes of a part of the genetic factors and the distributions of the trait values in the eight environments (MF and TF in 2018 and 2019) are presented in Supplementary Fig. S23.

The coefficients of the terms (G, E, and G×E) in addition to the constant term in the models (P = G + E + G×E) for the traits FT, *K*, and *r* (Supplementary Tables S16, S20, and S24) are visually presented in the bottom of Supplementary Fig. S22. In the FT model, for example, *Dt1*/*GmTFL1* showed a slight positive G×E interaction in latitude while a negative interaction in T_0_. In the *K* model, on the other hand, *Dt1*/*GmTFL1* had strong negative G×E interaction both in latitude and T_0_. In addition, T_30_ was positively interacted with *Dt1*/*GmTFL1* in the *K* model. In the *r* model, sowing time positively interacted with *Dt1*/*GmTFL1* and the other three environments indicated negative interaction. Interestingly, *E2* and *E3*, well-characterized genes for flowering, indicated completely opposite G×E interactions to each other in the FT model. Thus, the present models are informative to examine G×E interaction for flowering, plant height and initial growth of soybeans and further analysis on individual genetic factors would enable us to determine what G×E interaction contributes for the traits.

**Figure 4:**
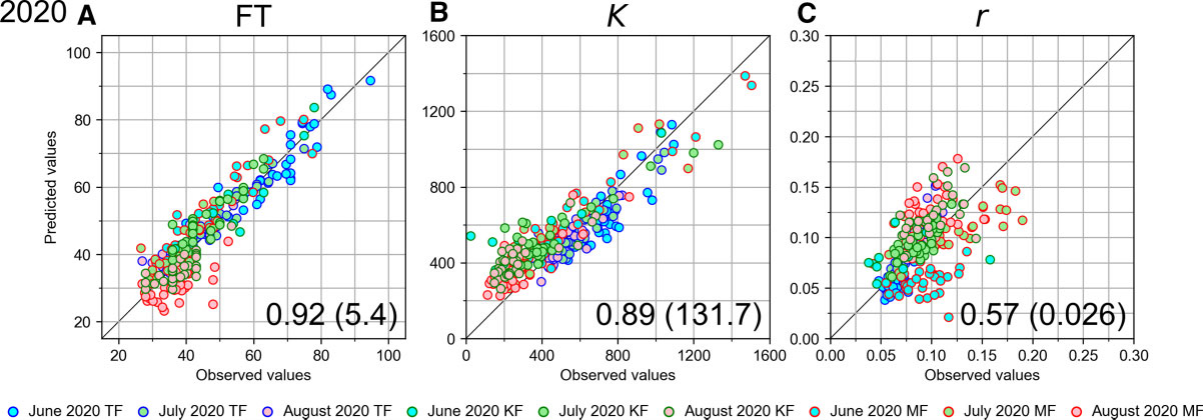
Scatter plots depicting the relationships between the observed values and the predicted values of the traits FT, *K*, and *r* calculated by the models (P = G + E + G×E) with genetic factors (50 sequence variants) and the environmental factors (average temperatures, sowing date, and latitude) and their interactions using the test data in 2020. (A) Relationship between the observed values and the predicted values in relation to the FT model; (B) relationship in relation to the *K* model; (C) relationship in relation to the *r* model. The horizontal axis and the vertical axis show the observed values and the predicted values, respectively. Samples in TF, KF, and MF are indicated by small circles with blue, green, and red edge colour. Samples observed in June, July, and August in 2020 are coloured in cyan, light green, and light red. The Pearson’s correlation coefficient between the observed values and the predicted values is indicated at the bottom right of each plot. The RMSE is also indicated in the parentheses.

For the evaluation, the relationship between the observed values and the predicted values in each field was compared using the samples observed in 2020. In relation to the three traits FT, *K*, and *r*, Fig. 4 shows the relationship between the observed values and the predicted values in the plot containing all the data in MF, TF, and KF. In the case of the FT model, the Pearson’s correlation coefficient (*R*) and the RMSE, respectively, became 0.92 and 5.4, indicating high predictability of the constructed model for the new data not only in the trained field but also the data from the novel field (Fig. 4A). In the plot of KF samples, a high prediction ability (*R *=* *0.91 and RMSE = 4.7) was obtained (Supplementary Fig. S24C). TF samples aligned on the diagonal line in the plot and a high prediction ability (*R *=* *0.97 and RMSE = 4.0) was obtained (Supplementary Fig. S24B). In the plot of MF samples, the August-sowing samples were underestimated and their predicted values were lower than observed values, resulting in a relatively low prediction ability (*R *=* *0.85 and RMSE = 6.8; Supplementary Fig. S24D). In the case of the *K* model, the correlation coefficient and the RMSE were, respectively, 0.89 and 131.7, indicating high predictability of the model even adding the data from the novel field (Fig. 4B). In the plot of KF samples (Supplementary Fig. S24G), the correlation coefficient and the RMSE, respectively, became 0.87 and 171.7 in spite of the tendency of higher prediction values compared with observed values. Regarding the *r* model, the correlation coefficient and the RMSE were, respectively, 0.57 and 0.026, indicating relatively low predictability of the model partly due to the tendency of higher prediction values compared with observed values in August-sowing samples (Fig. 4C). In the plot of KF samples, this tendency in the August-sowing samples was observed, resulting in a relatively low prediction ability (*R *=* *0.62 and RMSE = 0.024; Supplementary Fig. S24K). TF samples aligned on the diagonal line in the plot and a high prediction ability (*R *=* *0.92 and RMSE = 0.015) was obtained (Supplementary Fig. S24J). In the plot of MF, the August-sowing samples were overestimated and their predicted values were higher than observed values and the June- and July-sowing samples were underestimated and their prediction values were lower than observed values, resulting in a low prediction ability (*R *=* *0.44 and RMSE = 0.035; Supplementary Fig. S24L). The former tendency could have been caused by the high temperature in 2020 from mid-August to early September, which had not been experienced in 2018 and 2019. Other than the samples in MF, however, the samples including those in KF (i.e. in TF and KF) were aligned along the diagonal line and achieved a high prediction ability (*R *=* *0.77 and RMSE = 0.020), and thus it could be considered that the constructed model for *r* is basically applicable to the new data from the trained field and the novel field. Overall, it was confirmed that the constructed regression models for FT, *K*, and *r* were applicable to new data including a novel field.

## 4. Discussions

Crop growth simulation models have been widely studied to enable us to produce important crops stably under the environmental variations in climate change. However, the construction procedure of a robust model utilizing both genetic and environmental factors with their interactions (G×E) remains to be established. Here, we evaluated the effects of environments on the phenotype through field experiments of soybean core collections (93 cultivars) using multiple sowing times in multi-latitudinal fields. Using the high-density genetic markers obtained by re-sequencing of the collections and the detailed growth phenotypes under the diverse environments, we constructed multiple regression models targeting the quantitative traits including the FT and the plant height.

### 4.1. Contribution of environmental factors in soybean growth and development

As a result of multiple sowing time comparisons, the sowing time shifts from June to July and August shortened the time from sowing to flowering in both MF and TF. Among the tested soybean cultivars, ordering of the FT was not affected by sowing time shift in both MF and TF, indicating higher genotype contribution. Considering the environmental factors affected by sowing time shifts in our experiments, an increased temperature in the early growth phase and/or a decreased day length could be considered as the cause of shortening the time from sowing to flowering. The multiple sowing results obtained by Setiyono et al.,^32^ where the variation in sowing times from April to June lengthened the time from sowing to flowering, opposite to our result. In this condition, the temperature was increased depending on the sowing time shift similar to our experiment, while day length became longer depending on the sowing time shift opposite to our experiment. Therefore, the environmental factors that affected FT by the sowing time shifts could be considered as day length rather than temperature. Another field study confirmed that the sowing time shift caused the vegetative period to be shorter or longer depending on the photoperiod during the cultivation time.^33^^,^^34^ Thus, it is clear that sowing time shift affects FT based on the day length shift.

In soybean, it has been reported that a temperature range from 18°C to 26°C is ideal for growth and development.^35^ However, a study based on growth modelling by Boote^36^ predicted that 22–24°C is the optimum temperature for soybean growth. In the fields, a higher daily average temperature was observed in MF than in TF by 3–4°C, and this temperature difference might be reflected in the enhanced growth rate at the fourth week, when most of the cultivars were before flowering. This indicates that the higher temperature condition basically increases the growth rate, which might be explained by the improvement of leaf photosynthesis, since the direct target of temperature is the expansion of leaf area.^16^ Considering the lower temperature effect, on the other hand, a day average temperature <22°C was observed until mid-July in 2019 in TF (up to 7 weeks for June sowing), and resulted in the significant reduction of terminal plant height in comparison with June 2018 TF. This result supports the estimation by Boote,^36^ and a long period of day average temperature <22°C might affect the growth potential of soybean cultivars.

### 4.2. Environmental factors selected for the model construction

We selected T_0_ and T_30_ from the average temperatures of ten 10-day periods after the sowing (T_0_ to T_90_). T_0_ and T_30_ were selected in chronological order from the sowing under the condition that they were not correlated to each other in the training data obtained in the eight environments, meaning that the ones other than T_0_ and T_30_ were correlated with at least one of T_0_ and T_30_. If we loosen the threshold for the correlation (0.5 was used in this study), we could include the average temperatures of more periods. Although further optimization of the number of the periods in the models remained for the training data with more variety of meteorological conditions, the values of T_0_ and T_30_ could characterize the chronological patterns of the average temperatures, improving the prediction ability both by the environmental factors (E) and their interactions with genetic factors (G×E) as shown in Figs 3 and 4, Supplementary Figs S16–S18. In this way, T_0_ and T_30_ were selected without information of trait values, but the coefficients of the terms including them in the models were determined using the trait values in the training data. Therefore, if there existed the growth characteristics in response to temperature as above, the models were expected to represent those characteristics in a numerical manner.

The model construction process did not use any information indicating which environment the samples belong to. Since the parameters of the models were determined so that the estimated values and the observed values of all the samples were concordant with each other as a whole, specific samples sharing the same environmental condition could be found in the regions separated from the diagonal line. For example, in the Fig. 3D, H, and L, the samples were arranged in linear symmetry with respect to the diagonal line as a whole, however, we could find that several groups of samples were clustered and arranged in the region either over or under the diagonal line. This is one of the reasons why the prediction accuracy in a specific field remained low (Supplementary Fig. S24). This separation was partly caused by the baseline values determined by the environmental factors (E). We could observe that the ordering of the samples in the groups was properly estimated and predicted (Figs 3 and 4). This result indicated the applicability of the developed model was evaluated in new environments (i.e. in 2020 including KF). As we used the same set of the soybean accessions in 2020, evaluation of the applicability of the developed model for new combinations of genetic factors would deserve further investigation.

### 4.3. Genetic factors selected in the multiple regression models

By performing GWAS separately in each field and sowing time, the peaks of Manhattan plots for a given trait showed different patterns depending on the field and sowing time conditions. For the variants associated with FT, two major genes *E2* and *E3* were detected in almost all conditions with a field-dependent manner. Although *E2* and *E3* were detected as the top peaks in TF, they were not detected as the top peaks in MF. Instead, *Glyma.12G073900* was detected as a top peak for June and July in MF, which encodes pseudo-response regulator protein reported to be interrelated with *E2*.^37^ In the association with plant height, *Dt1/GmTFL1* was significantly detected in all eight environmental combinations, yet the peak also showed different pattern across environments. These results indicate that these genetic factors might have interaction with the environmental effect or called G×E.

Indeed, as the result of genetic factors selected from the multiple regression model, in which the effect of G×E was considered, those major genes were found to significantly contribute to the model estimation. For example, under the interaction effect of *Dt1/GmTFL1* and temperature (T_30_) in the FT model (Supplementary Fig. S22), the coefficient appeared to be positive indicating that the function of *Dt1/GmTFL1* regarding FT control could be enhanced under warm growing conditions. This result is consistent with the study by Ogiso-Tanaka et al.^38^ who found that *Dt1*/*GmTFL1* was significantly associated with FT in the population grown in Tsukuba in 2013, when the temperature was relatively higher during early growing period, but not in 2011 which had lower temperature condition.^38^ Moreover, the selection of *Dt1/GmTFL1* in the FT model reflects its pleiotropic effects in regulating multiple traits as reported in the previous studies.^39–42^ Yue et al.^43^ have investigated the role of *Dt1/GmTFL1* to regulate not only plant height but also FT in soybean by its interaction with a transcription factor FDc1 to repress the expression of *APETALA1* (*AP1*).^43^

Taking advantage of the genetic factors selected in our constructed models, we identified alleles on the genes that have been reported to play important roles in plant growth and development. For genetic factors in the *K* model, *Glyma.04G179400* encodes the homolog of *Arabidopsis DCC1*, which has been reported to be involved in shoot development. *DCC1* encodes thioredoxin that interacts with carbonic anhydrase 2 to regulate mitochondrial complex I activity, and mutation of this gene led to low shoot regeneration in *Arabidopsis* callus.^44^*Glyma.13G136700* encodes the homolog of *Arabidopsis* LRR receptor kinase, *AT2G37050*, which has been identified highly accumulated in the *Arabidopsis* root and proposed to be involved in root development by regulating the expression of the CLASP family protein^45^ that involved in promoting microtubule stability.^46^ Another allele on *Glyma.12G076900* encodes the homolog of *Arabidopsis* RRP41L protein, which is predicted as 3′-5′-exonuclease involved in cytoplasmic mRNA decay.^47^ Mutation on *RRP41L* has been shown to affect early seedling growth such as delayed germination and reduced early growth rate in *Arabidopsis*.^47^*Glyma.09G179600* encodes the homolog of *Arabidopsis* membrane-associated M1 protease (APM1), which has been suggested to be involved in the cell division, root meristem development, and vascular tissue maintenance by interacting with transcription factors PLETHORA and BABY BOOM.^48^ Loss-of-function mutants of *APM1* resulted in root growth defect in *Arabidopsis*.^48^*Glyma.03G140800* encodes the homolog of *Arabidopsis PLT2*, which has been reported to regulate biosynthesis and transport of auxin to coordinate growth and differentiation,^49^ as well as to specify stem cell niche^50^ in *Arabidopsis* root. *Glyma.05G166400* encodes the homolog of *Arabidopsis* homeodomain–leucine zipper containing protein, ATHB14, which is involved in the determination of adaxial-abaxial polarity in leaf and ovule primordium.^51^

For genetic factors in the *r* model, several genes related to organ development were selected. For instance, *Glyma.19G118900* encodes the homolog of *Arabidopsis* VAR3 zinc-finger protein, which involves in chloroplast and palisade cell development.^52^*Glyma.10G143600* encodes the homolog of *Arabidopsis* LCD1, which reported to be involved in internal leaf formation, altering mesophyll cell arrangement.^53^ In addition, recent study shows that mutation in *LCD1* caused delay of leaf senescence revealed by reduction of chlorophyll degradation in tomato leaves.^54^ It was assumed that LCD1 delays the chlorophyll degradation by regulating key genes in the chlorophyll degradation pathway and senescence-associated genes.^54^*Glyma.01G040900* encodes a putative DExH box RNA helicase, the homolog of *Arabidopsis* ESP3 (AT1G32490). ESP3 has been investigated to play a role in controlling cell wall-related proteins, such as peroxidases and xyloglucan endotransglucosylases.^55^*Glyma.15G008000* encoding the homolog of *Arabidopsis* epidermal patterning factor-Like 8, *EPFL8*, has been reported to regulate ERECTA family receptors with the role in the communication of leaf boundary and central zone of SAM.^56^

In this study, we carried out detailed phenotyping of the cultivars with a wide range of genotypes under a variety of environments created by multiple sowing times in two fields. By analysing the obtained phenotype data, it can be considered that day length and temperature are the main contributors for phenotypic plasticity in soybean cultivars. GWAS for the obtained phenotypes showed the changing effects of individual genetic loci in different fields and sowing time indicating the effect of G×E. Based on accumulated phenotypic data, multiple regression models targeting the FT and plant height were constructed. The applicability of the constructed models was confirmed by the field experiments data in 2020 including a new field. As the constructed models were composed of genetic factors including known genetic loci, temperatures in growth period as environmental factors, and their interactions, it is feasible to simulate the dynamics of growth pattern under a given environmental condition. Therefore, the data set obtained in this study would contribute to sustainable soybean cultivation in the future climate change.

## Supplementary Material

dsac024_Supplementary_Data
