# Supplementary material for: Construction of prediction models for growth traits of soybean cultivars based on phenotyping in diverse genotype and environment combinations
Source: DNA Res. 2022 Aug 2;29(4):dsac024. doi: 10.1093/dnares/dsac024 (PMC9358015; doi:10.1093/dnares/dsac024)
Supplement: dsac024_Supplementary_Data [file dsac024_supplementary_data.zip › dsac024_Supplementary_Data/Supplementary_Figures_DNAR-2021-081_final.docx]

A Flowering time in TF

| 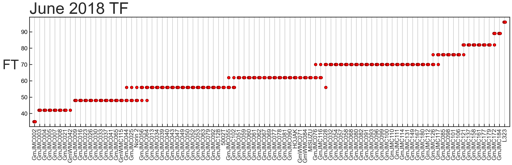 | 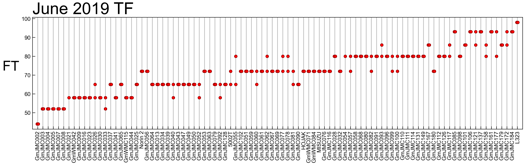 |
| --- | --- |
| 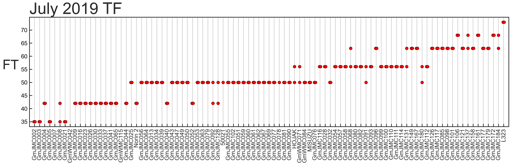 | 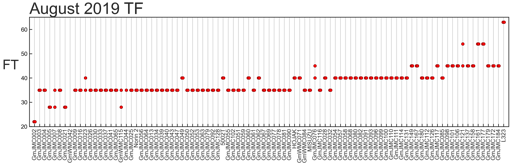 |

B Flowering time in MF

| 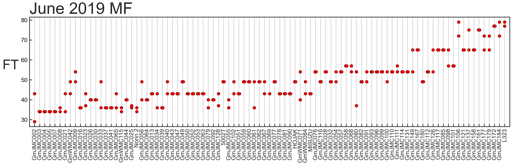 | 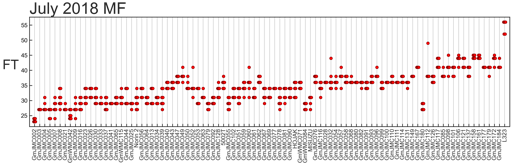 |
| --- | --- |
| 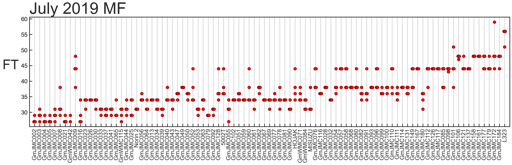 | 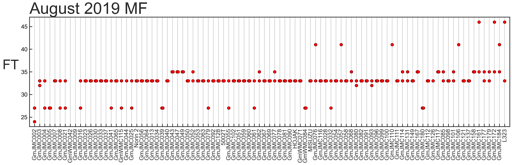 |

C Terminal plant height in TF

| 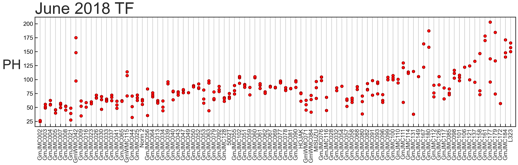 | 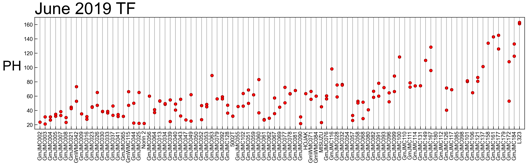 |
| --- | --- |
| 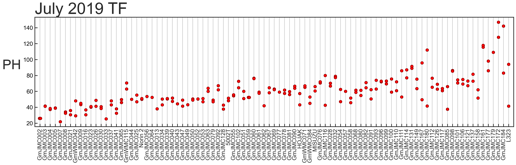 | 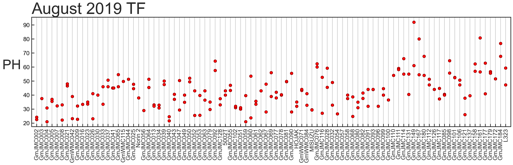 |

D Terminal plant height in MF

| 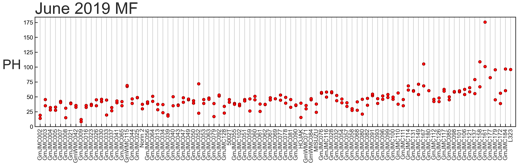 | 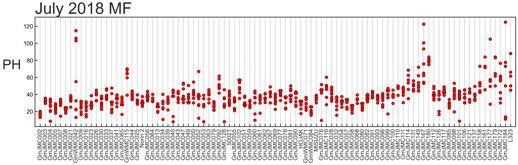 |
| --- | --- |
| 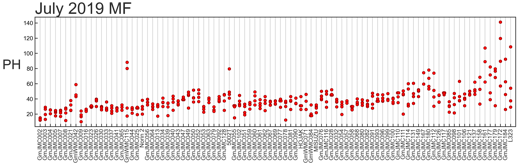 | 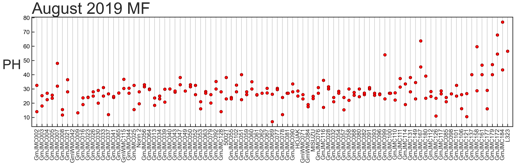 |

**Supplementary figure S1.** Distributions of the trait values of the individuals with replications in relation to each of the four sowing times in TF and MF. A, flowering time in TF; B, flowering time in MF; C, terminal plant height in TF; D, terminal plant height in MF; The vertical axis shows the trait values. The 93 cultivars were arranged along the horizontal axis in the increasing order of the flowering time in June 2018 TF.

**
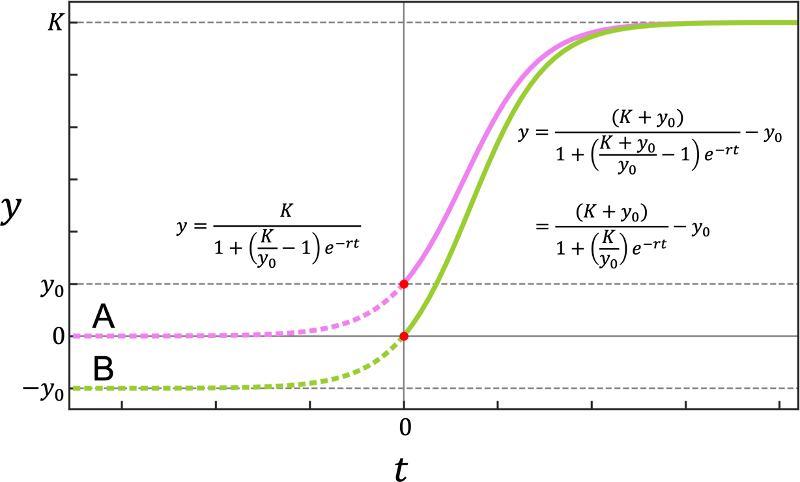
**

**Supplementary figure S2.** Definition of the function for growth curves of the plants. A, Values of a logistic function using the standard definition (magenta). B, Values of the logistic function we used (light green). The definition was changed so that the *y*-intercept of the curve could be 0 (*i.e.*, *y* = 0 when *t* = 0). The region represented by a solid line was used for fitting to the time series of plant height.


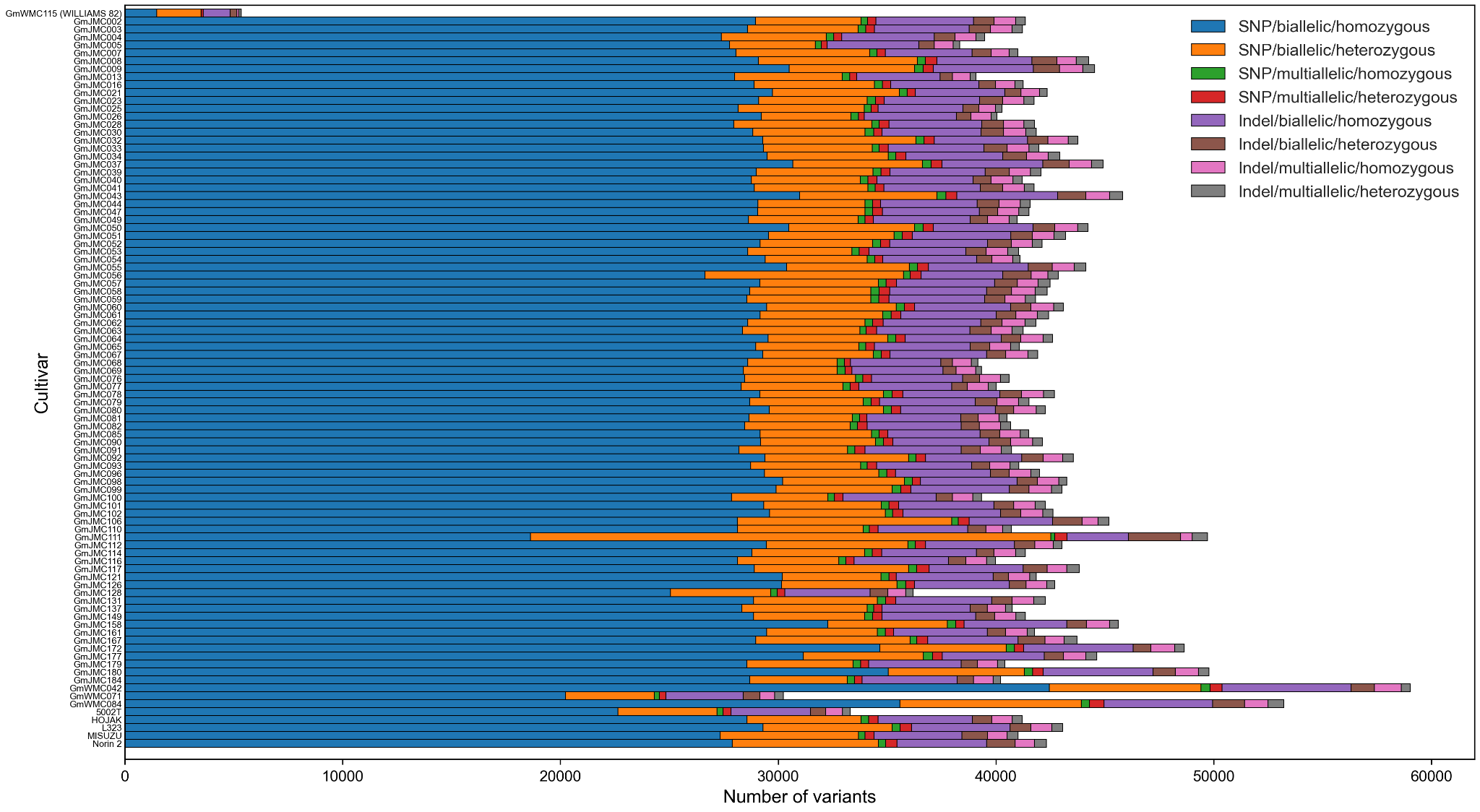


**Supplementary figure S3.** Number of the variants in the 93 cultivars. Stacked bars show the breakdown of the number of the variants at the 207,944 chromosomal positions extracted from the ‘initial-step’ variant dataset (dataset 1)^20^ in relation to whether they are SNPs or indels, biallelic loci with a single variant allele or multiallelic loci with multiple variant alleles, and homozygous or heterozygous.

| A | 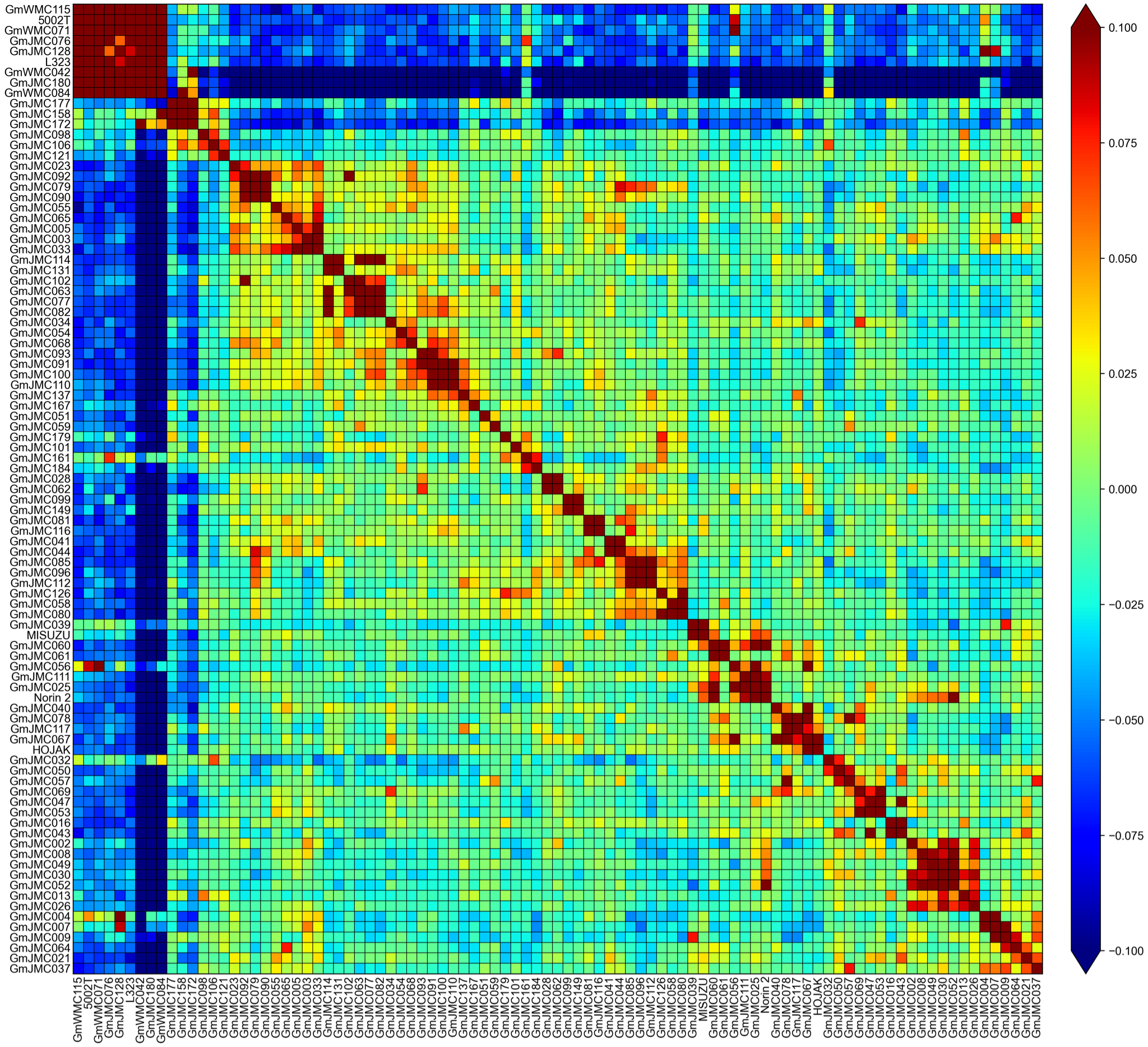 |
| --- | --- |
| B | 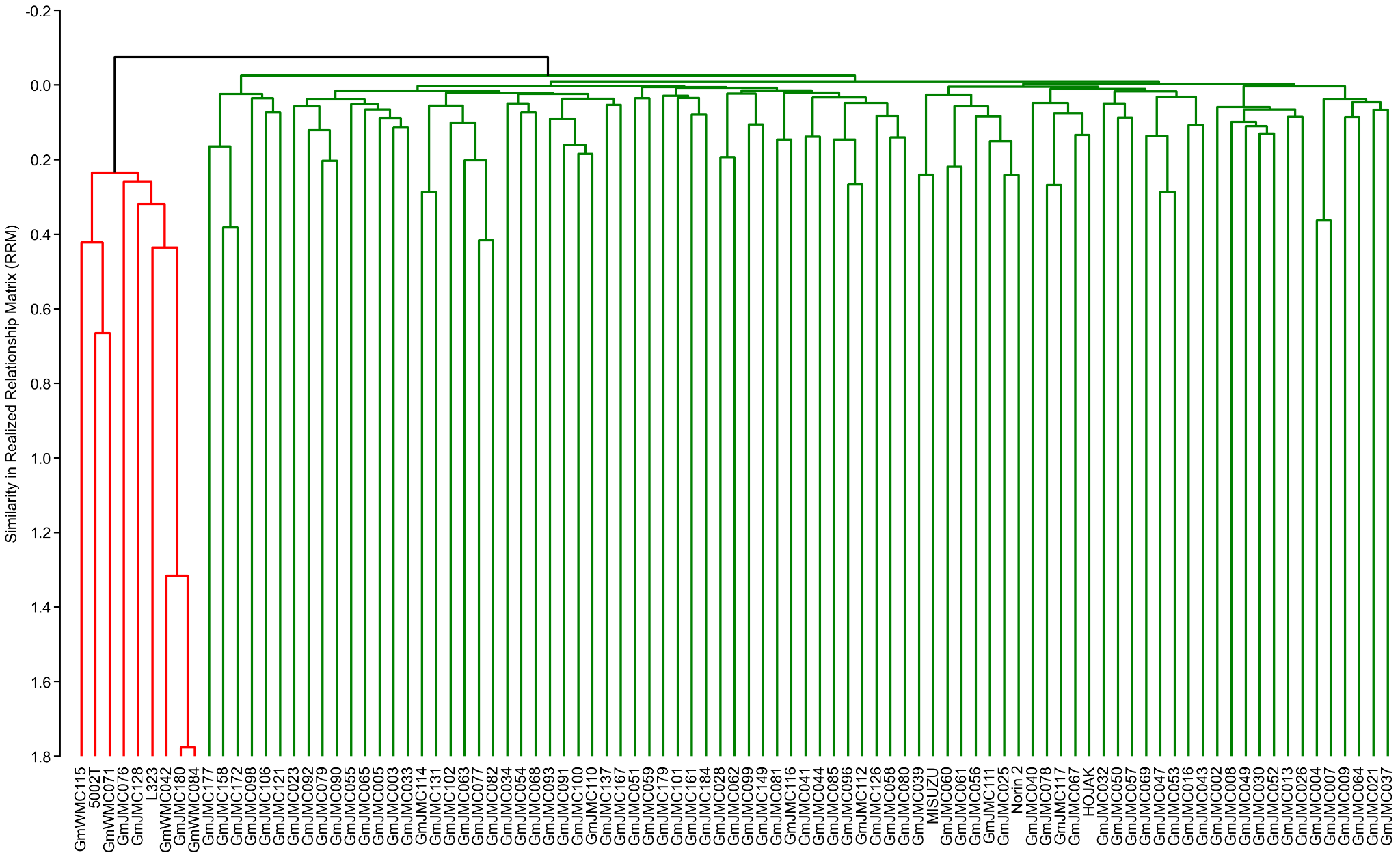 |

**Supplementary figure S4.** Genetic relationship among the 93 cultivars. A, Visualization of the relationship matrix among the 93 cultivars calculated by the ‘realized_relationship_matrix’ function of the Hail library (version 0.2.93) for the Python language. The names of the cultivars along the horizontal axis and the vertical axis were aligned according to the order obtained by the clustering result in B; B, A phylogenetic tree of the 93 cultivars. The dendrogram was generated by the hierarchical clustering (average-linkage method) based on the values in the relationship matrix in A. The top level branch of the dendrogram yielded two clusters in red and green. The red cluster contained all of the six cultivars from world collections used in this study (GmWMC115, GmWMC071, GmWMC042, GmWMC084, 5002T and L323), and three Japanese cultivars (GmJMC076, GmJMC128 and GmJMC180).


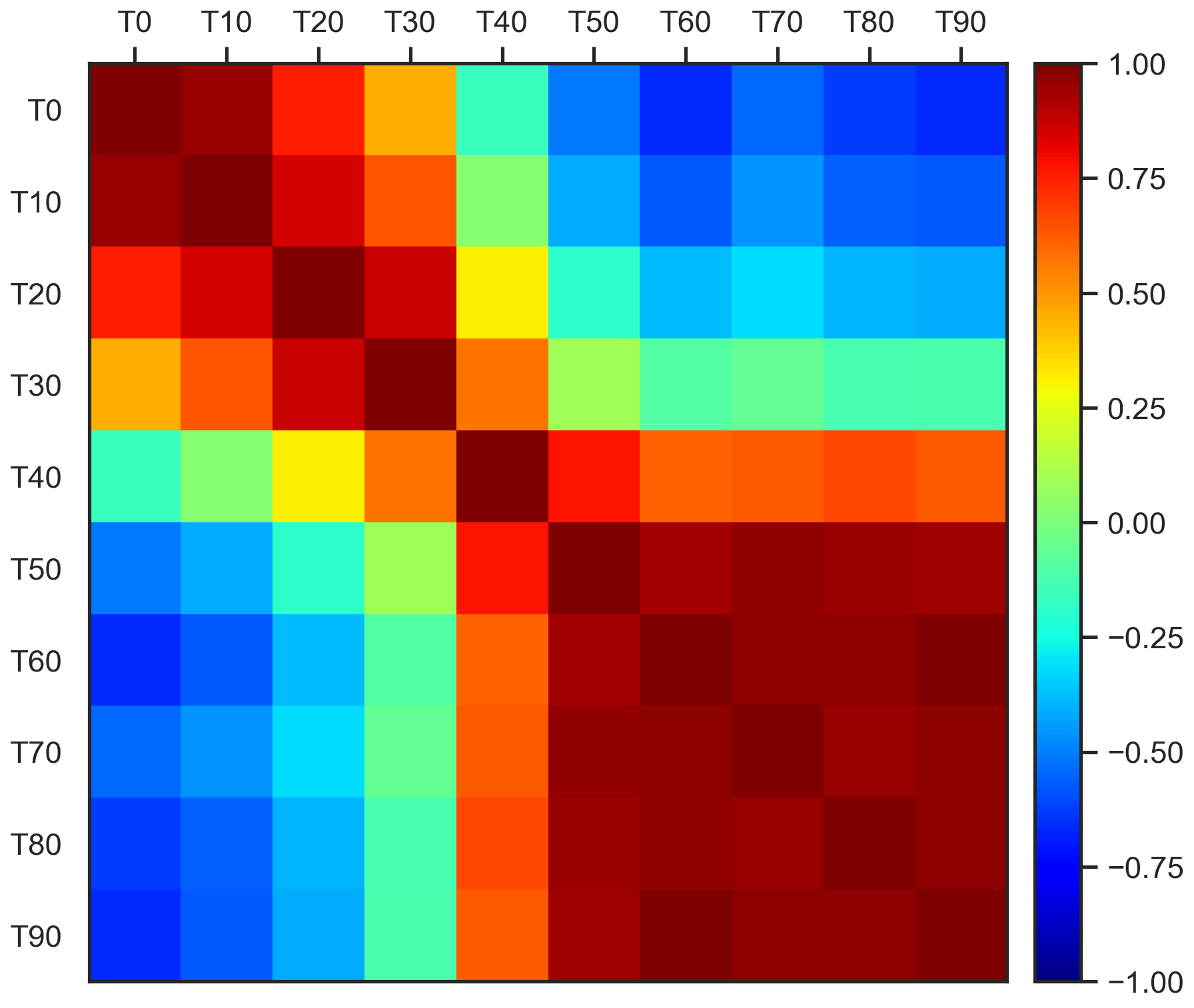


**Supplementary figure S5.** Heat map of the correlation matrix among T0 to T90 in the training data. Correlation between a pair of the average temperatures of periods were calculated by the patterns of the values in the eight environments.


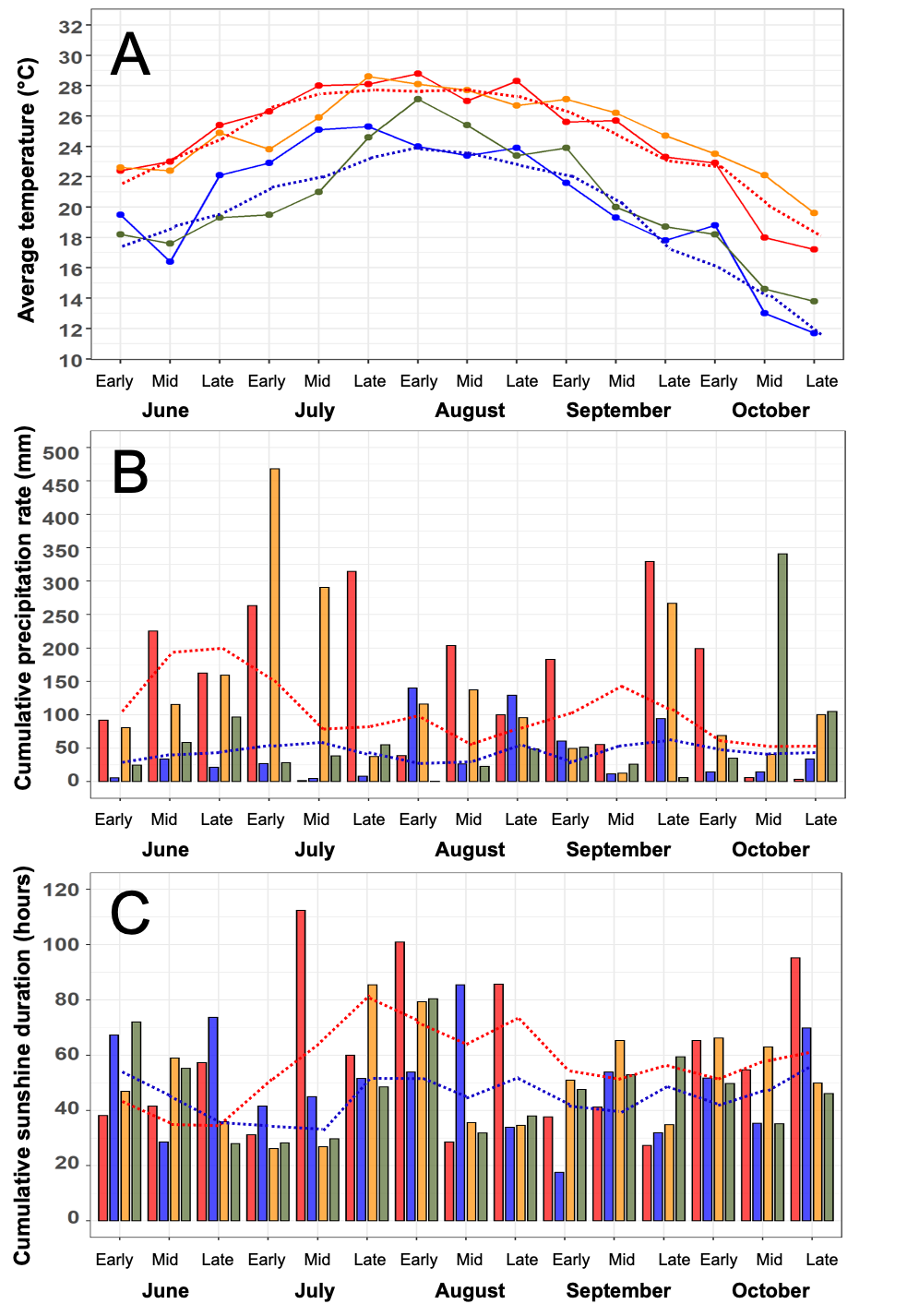


**Supplementary figure S6.** Environmental variations across fields and years during the growing period. A, Ten-days average temperature (°C); B, Ten-days cumulative precipitation (mm); C, Ten-days cumulative sunshine duration (hours). Red and blue colors represent MF and TF in 2018, respectively. Orange and green colors represent MF and TF in 2019, respectively. Dotted lines represent the 30 years average (from 1991-2020) in MF (red), and TF (blue).

**
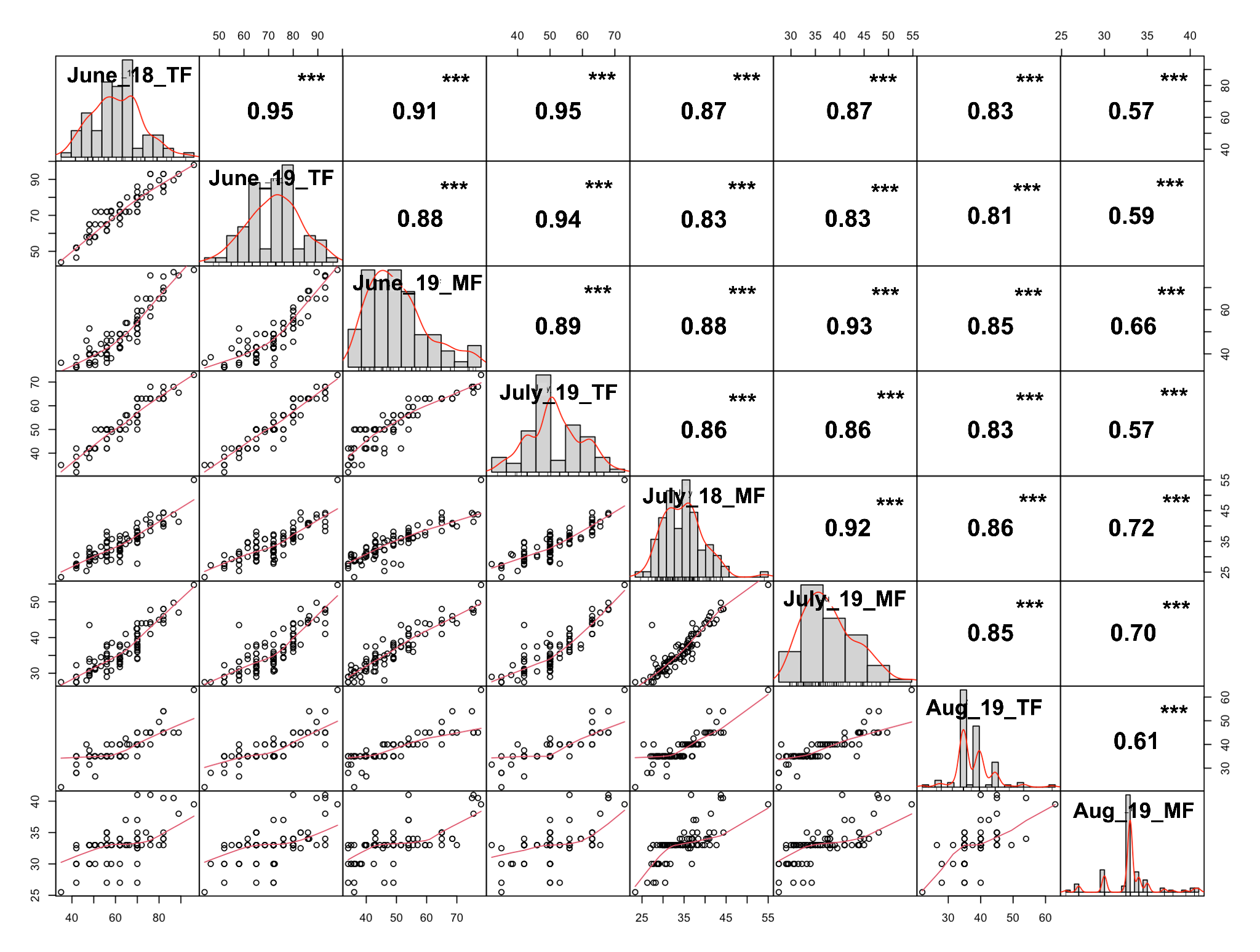
**

**Supplementary figure S7.** The correlation matrix of flowering time across eight environments. The top right of the figure indicated the correlation coefficients based on Pearson’s correlation while the bottom left part showed scatterplots with the fitted line. Significant differences are indicated by *** *p* < 0.001.

**
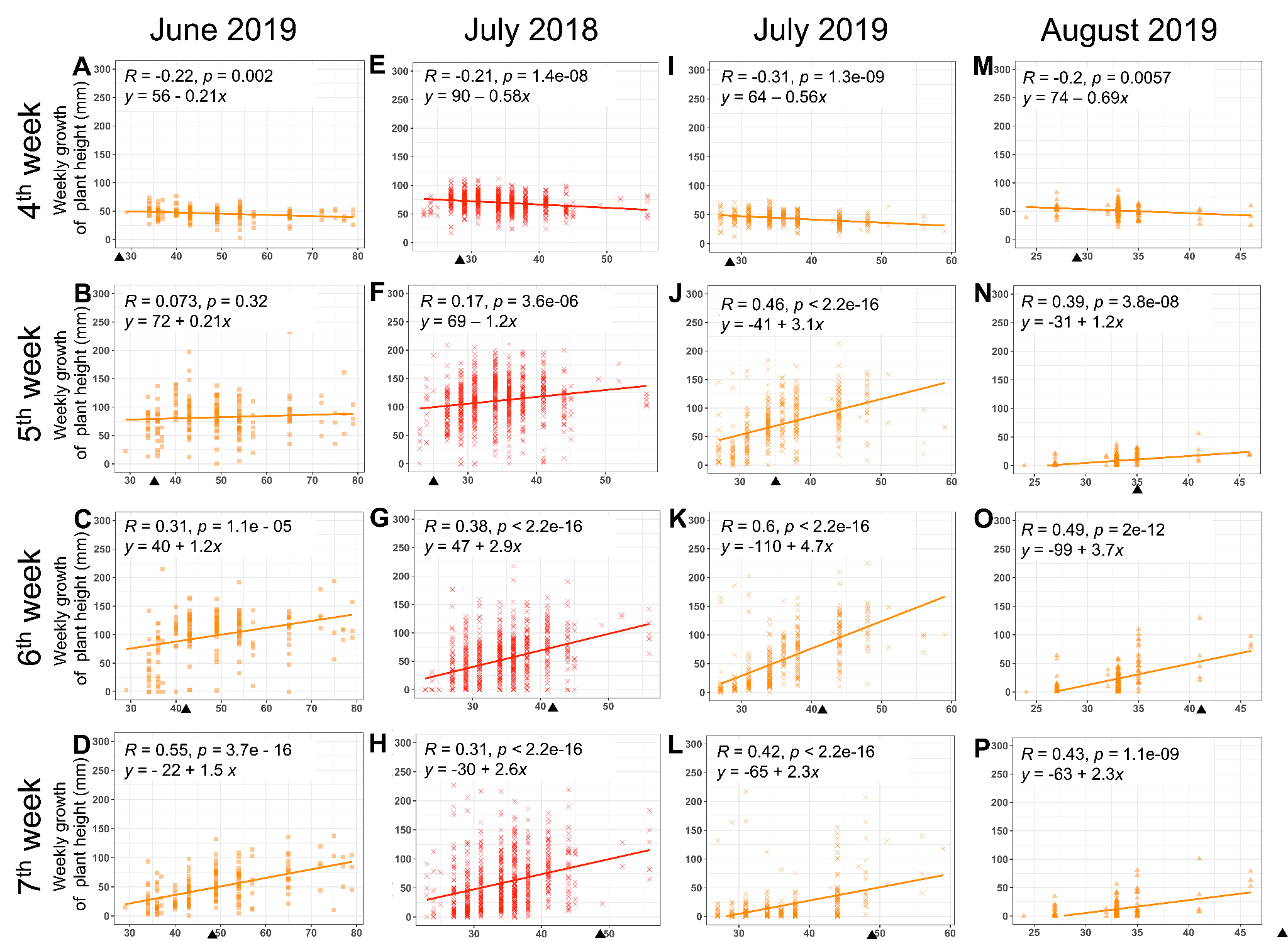
**

**Supplementary figure S8.** Relationship between flowering time and weekly growth of plant height (mm) in MF. A-D, sowing in June 2019 (182 individuals); E-H, sowing in July 2018 (685 individuals); I-L, sowing in July 2019 (174 individuals); M-P, sowing in August 2019 (177 individuals). A, E, I, and M, weekly growth based on seven days growth at 4^th^ week (growth from 3^rd^ to 4^th^ week); B, F, J and N, weekly growth at 5^th^ week; C, G, K and O, weekly growth at 6^th^ week; D, H, L and P, weekly growth at 7^th^ week. The correlation and *p* value were calculated based on Pearson’s correlation. A small triangle on the X-axis indicates the time point of evaluation.


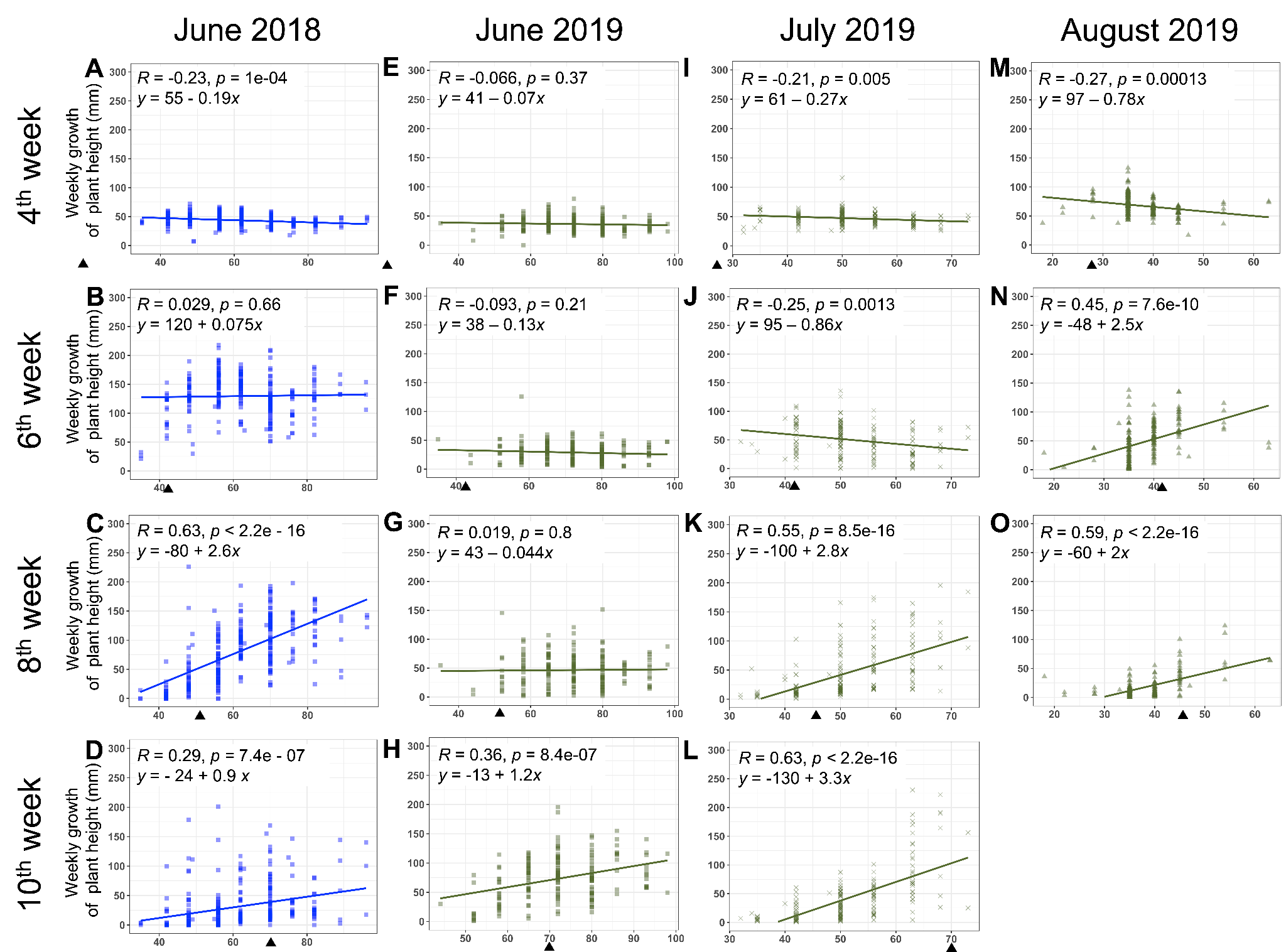


**Supplementary figure S9.** Relationship between flowering time and weekly growth of plant height (mm) in TF. A-D, sowing in June 2018 (245 individuals); E-H, sowing in June 2019 (143 individuals); I-L, sowing in July 2019 (174 individuals); M-O, sowing in August 2019 (168 individuals). A, E, I, and M, weekly growth based on seven days growth at 4^th^ week (growth from 3^rd^ to 4^th^ week); B, F, J and N, weekly growth at 6^th^ week; C, G, K and O, weekly growth at 8^th^ week; D, H, L and P, weekly growth at 10^th^ week. The correlation and *p* value were calculated based on Pearson’s correlation. A small triangle on the X-axis indicates the time point of evaluation.


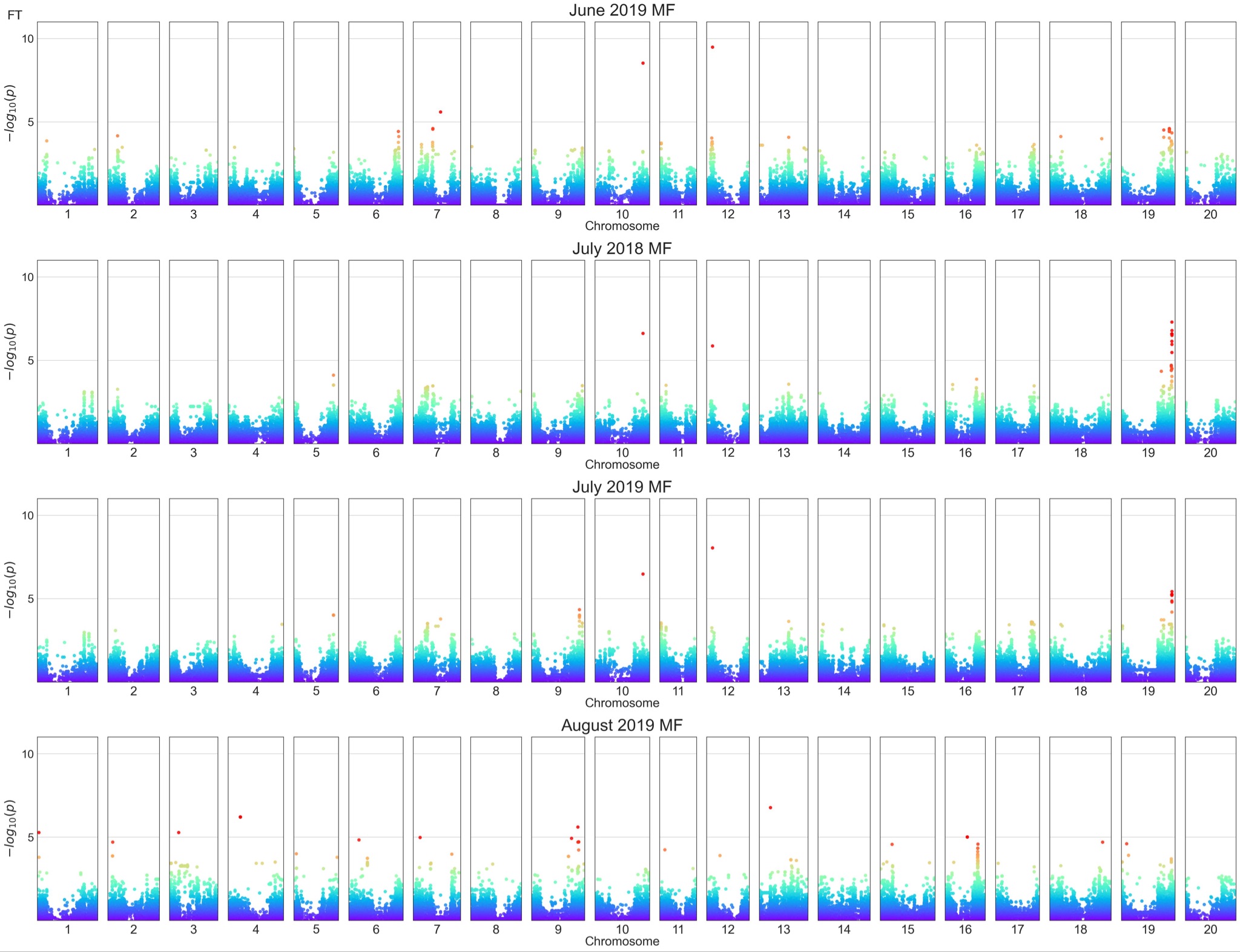


**Supplementary figure S10.** Manhattan plots of GWAS for flowering time in MF for three sowing times in 2018 and 2019. The X-axis shows chromosomal position. Y-axis represents -log_10_ *p* values. Chromosomal positions were omitted from the plot when the number of the cultivars that had the homozygous alternate genotype (1/1) was less than two or the number of the cultivars that did not have the homozygous alternate genotype (0/0 or 1/0) was less than two.

**
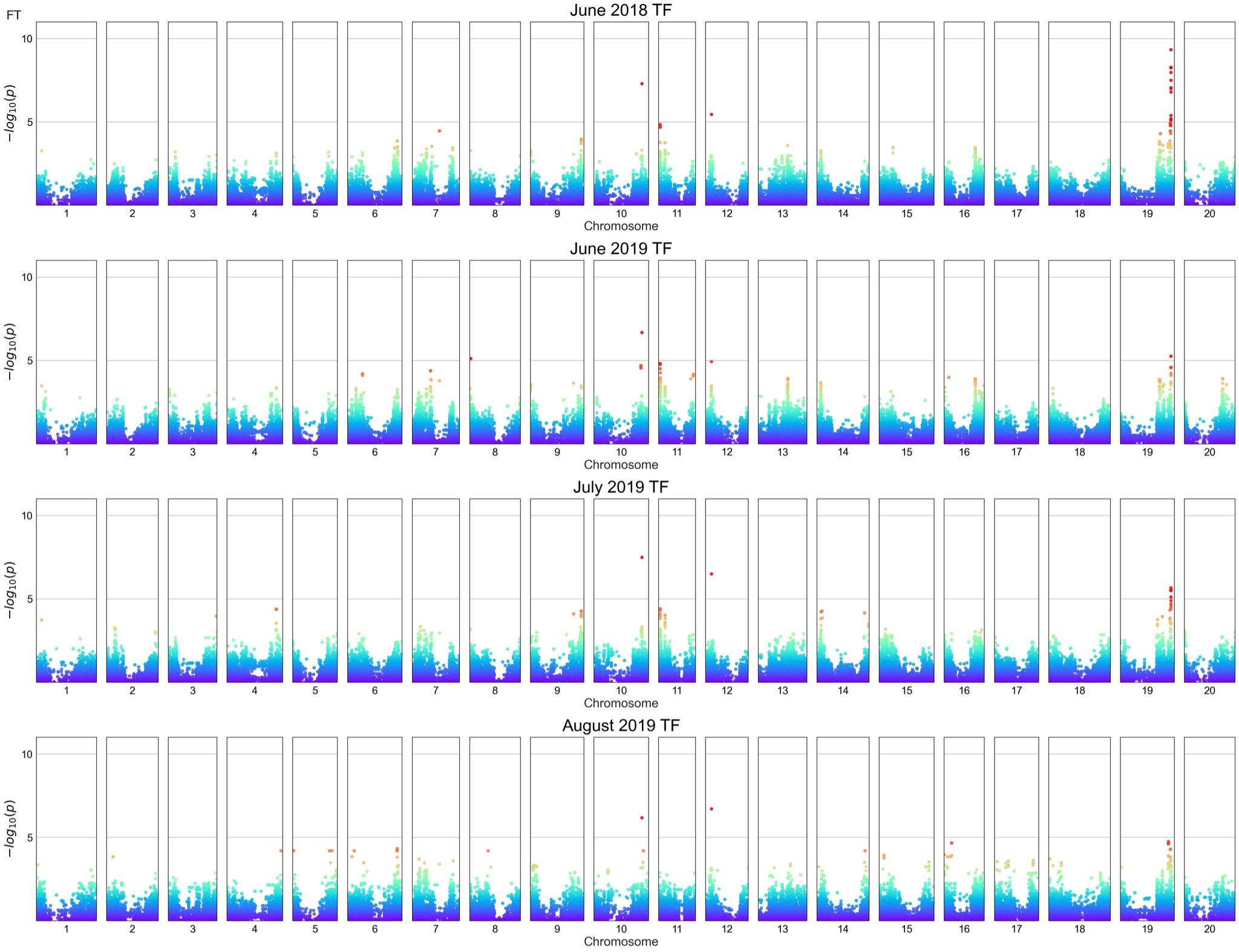
**

**Supplementary figure S11.** Manhattan plots of GWAS for flowering time in TF for three sowing times in 2018 and 2019. The X-axis shows chromosomal position. Y-axis represents -log_10_ *p* values. Chromosomal positions were omitted from the plot when the number of the cultivars that had the homozygous alternate genotype (1/1) was less than two or the number of the cultivars that did not have the homozygous alternate genotype (0/0 or 1/0) was less than two.


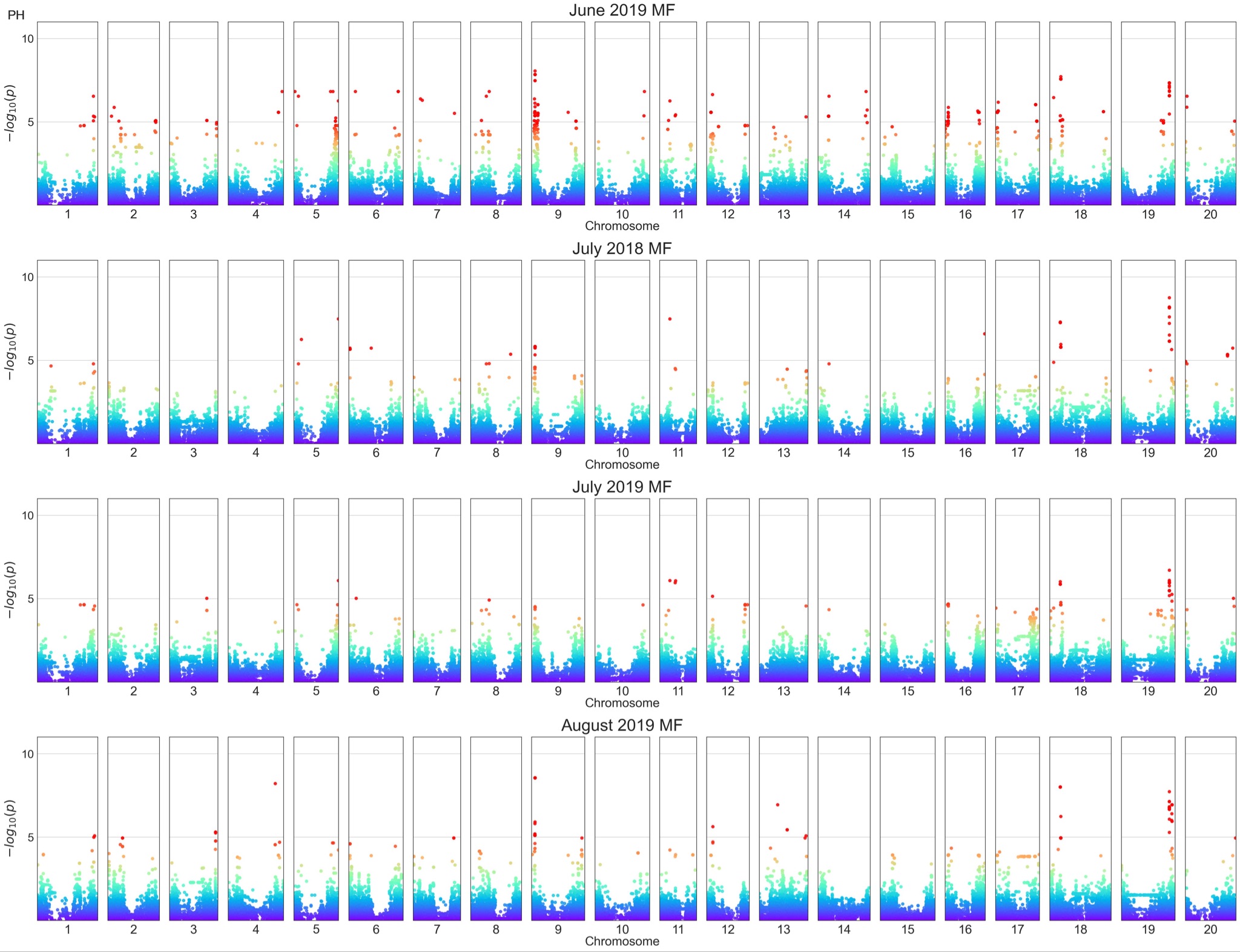


**Supplementary figure S12.** Manhattan plots of GWAS for plant height in MF for three sowing times in 2018 and 2019. The X-axis shows chromosomal position. Y-axis represents -log_10_ *p* values. Chromosomal positions were omitted from the plot when the number of the cultivars that had the homozygous alternate genotype (1/1) was less than two or the number of the cultivars that did not have the homozygous alternate genotype (0/0 or 1/0) was less than two.

**
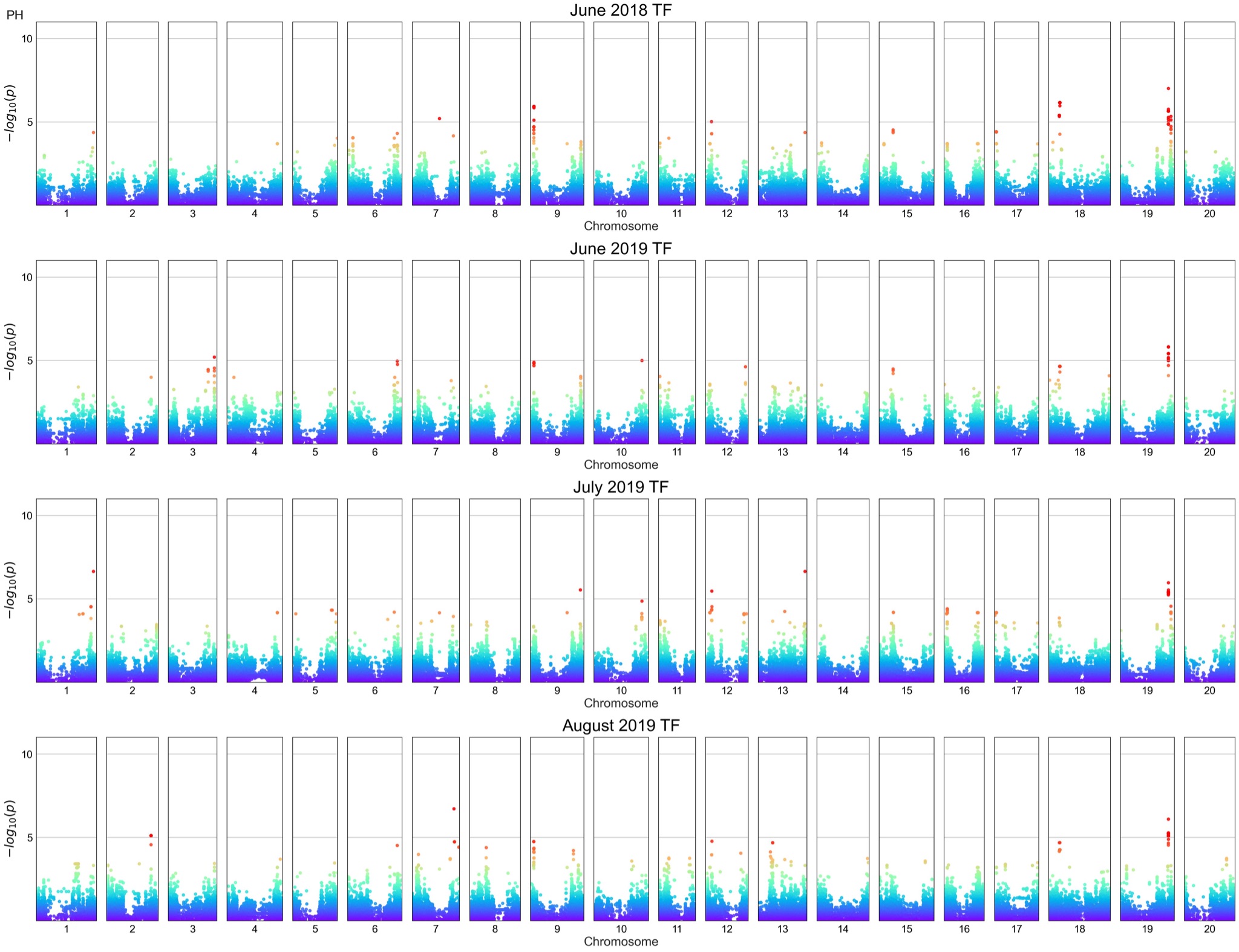
**

**Supplementary figure S13.** Manhattan plots of GWAS for plant height in TF for three sowing times in 2018 and 2019. The X-axis shows chromosomal position. Y-axis represents -log_10_ *p* values. Chromosomal positions were omitted from the plot when the number of the cultivars that had the homozygous alternate genotype (1/1) was less than two or the number of the cultivars that did not have the homozygous alternate genotype (0/0 or 1/0) was less than two.


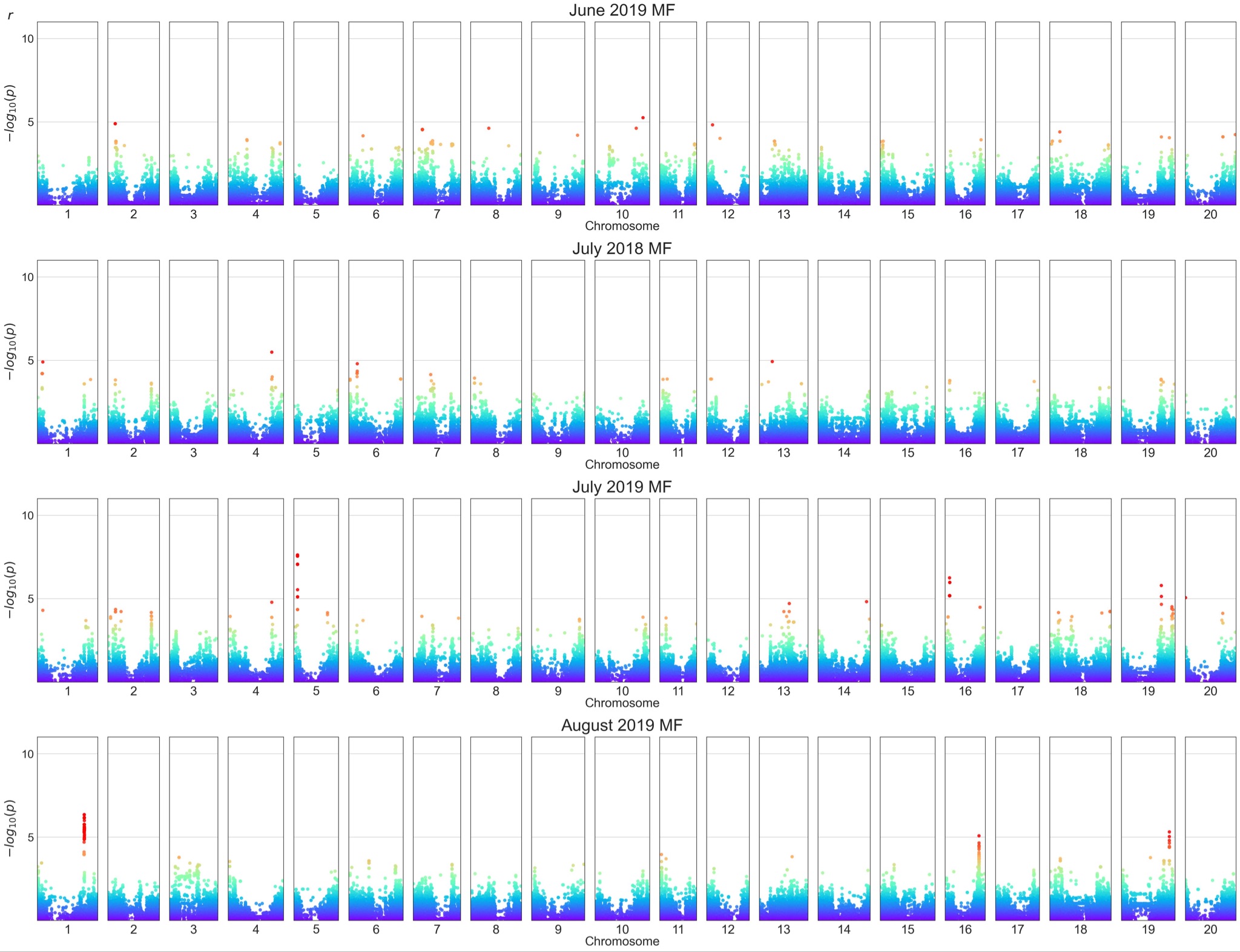


**Supplementary figure S14.** Manhattan plots of GWAS for *r* in MF for three sowing times in 2018 and 2019. The X-axis shows chromosomal position. Y-axis represents -log_10_ *p* values. Chromosomal positions were omitted from the plot when the number of the cultivars that had the homozygous alternate genotype (1/1) was less than two or the number of the cultivars that did not have the homozygous alternate genotype (0/0 or 1/0) was less than two.

**
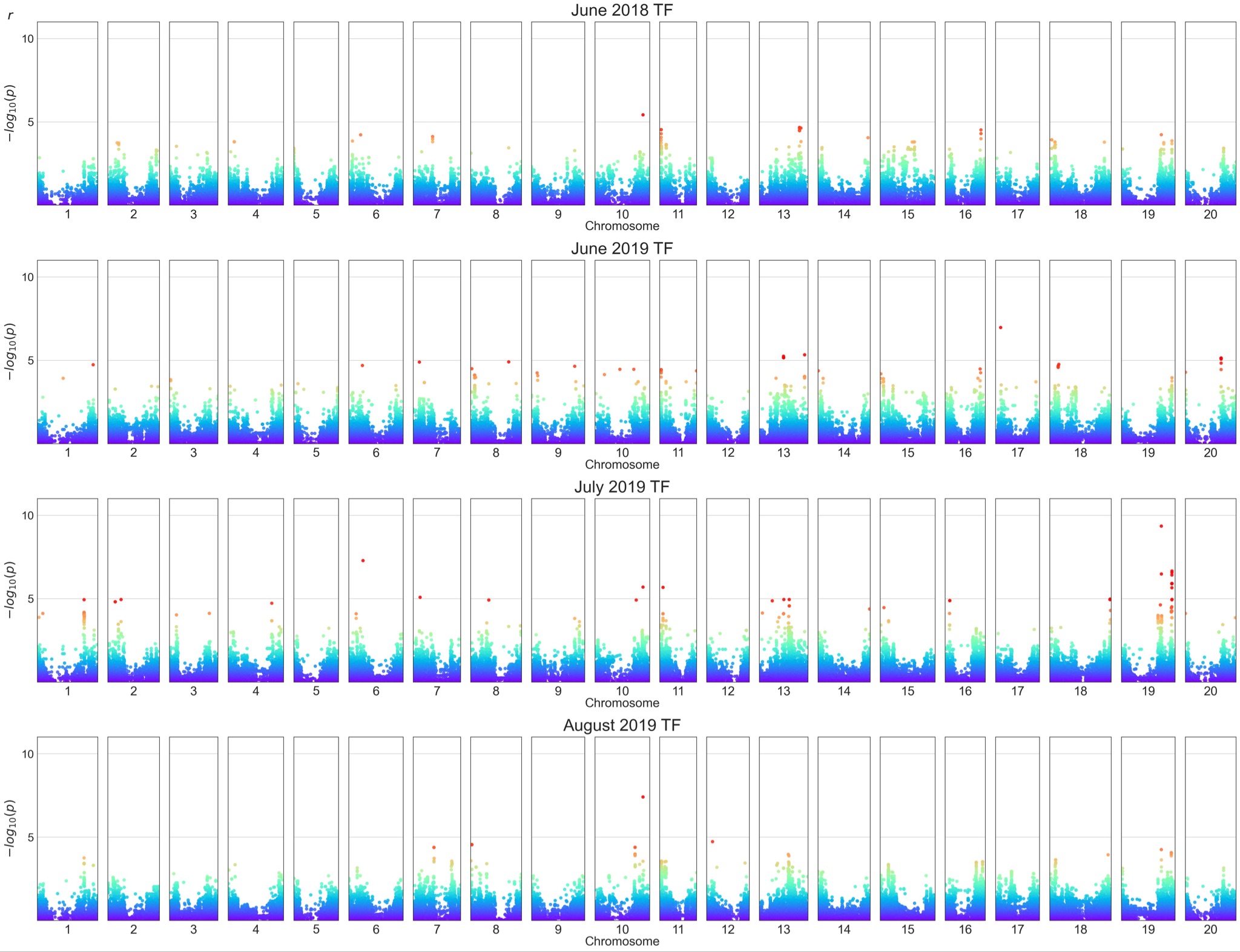
**

**Supplementary figure S15.** Manhattan plots of GWAS for *r* in TF for three sowing times in 2018 and 2019. The X-axis shows chromosomal position. Y-axis represents -log_10_ *p* values. Chromosomal positions were omitted from the plot when the number of the cultivars that had the homozygous alternate genotype (1/1) was less than two or the number of the cultivars that did not have the homozygous alternate genotype (0/0 or 1/0) was less than two.

**
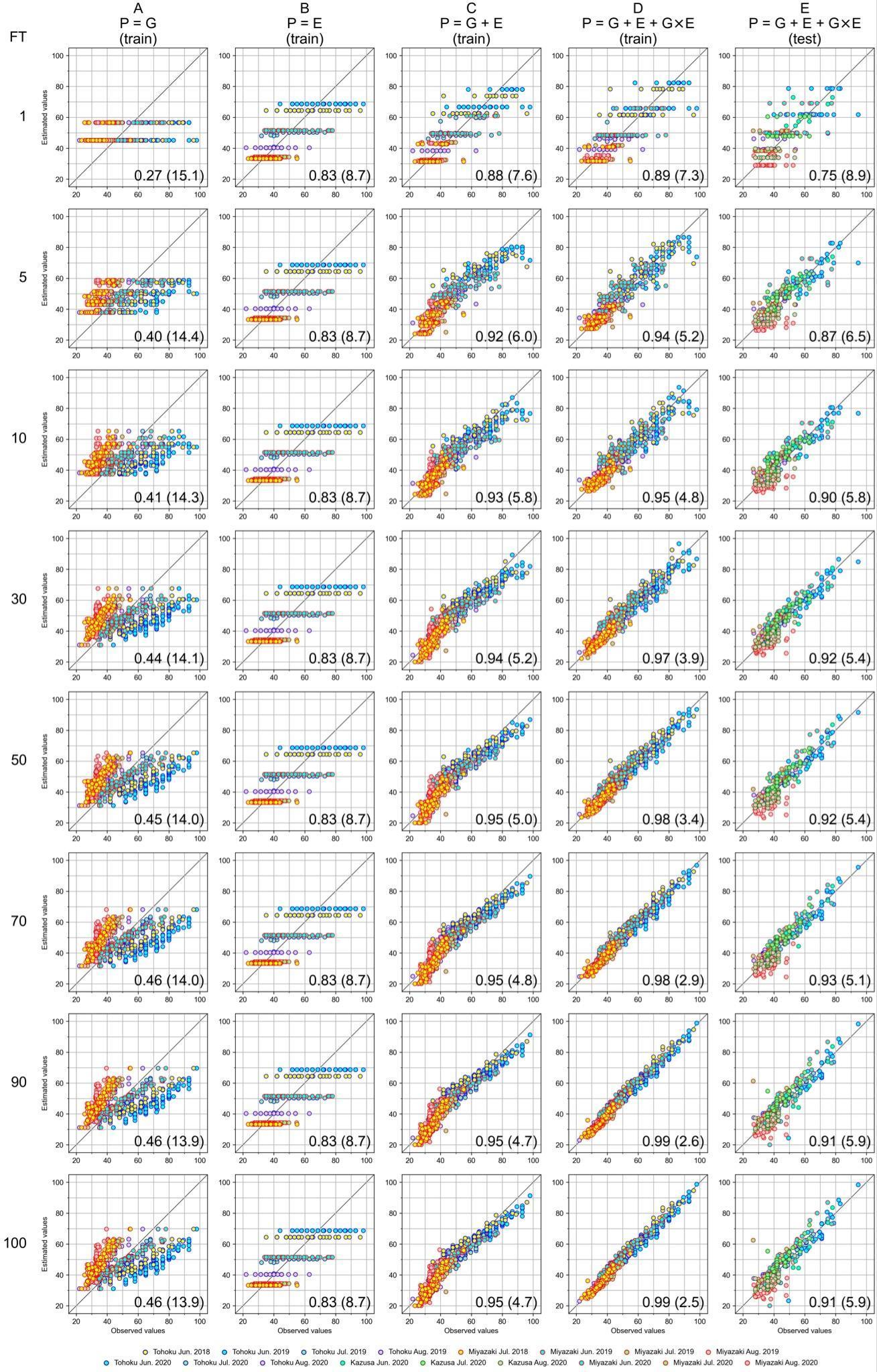
**

**Supplementary figure S16.** Relationship between the complexity of the models and the estimated and predicted values in relation to the FT model. A, P = G; B, P= E; C, P = G + E; D, P = G + E + GxE; A-D are fitting results in the training data (TF and MF in 2018 and 2019); E, the prediction results in the test data (TF, KF, and MF in 2020) by the model with P = G + E + GxE generated in D; The horizontal axis shows the observed values while the vertical axis shows the estimated values (A-D) or predicted values (E). Results with the 1, 5, 10, 30, 50, 70, 90, and 100 genetic factors are compared in the vertical direction. The Pearson’s correlation coefficient between the observed values and the estimated and/or predicted values is indicated at the bottom right of each plot. The root mean squared error (RMSE) is also indicated in the parentheses.

**
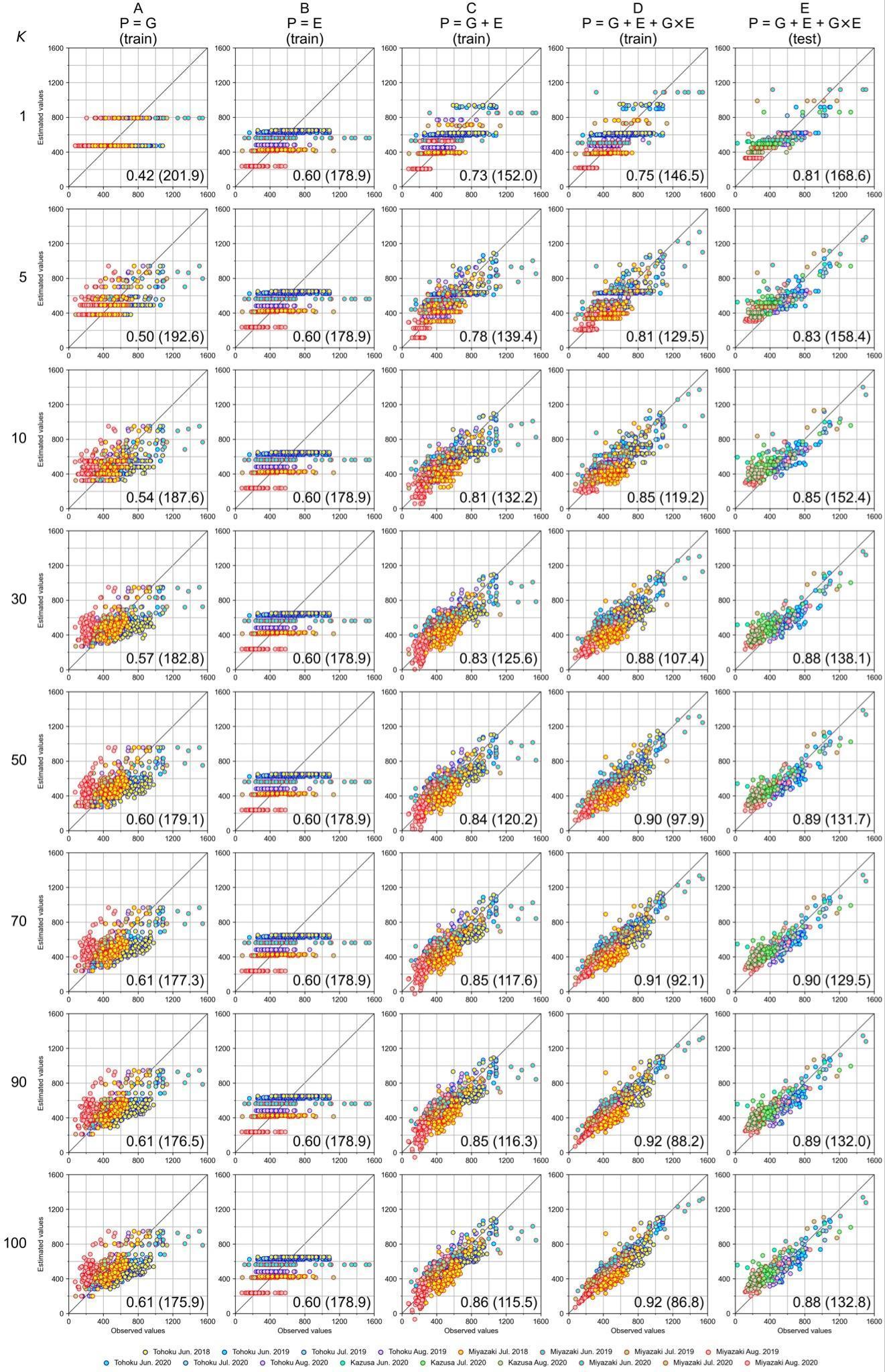
**

**Supplementary figure S17.** Relationship between the complexity of the models and the estimated and predicted values in relation to the *K* model. A, P = G; B, P= E; C, P = G + E; D, P = G + E + GxE; A-D are fitting results in the training data (TF and MF in 2018 and 2019); E, the prediction results in the test data (TF, KF, and MF in 2020) by the model with P = G + E + GxE generated in D; The horizontal axis shows the observed values while the vertical axis shows the estimated values (A-D) or predicted values (E). Results with the 1, 5, 10, 30, 50, 70, 90, and 100 genetic factors are compared in the vertical direction. The Pearson’s correlation coefficient between the observed values and the estimated and/or predicted values is indicated at the bottom right of each plot. The root mean squared error (RMSE) is also indicated in the parentheses.


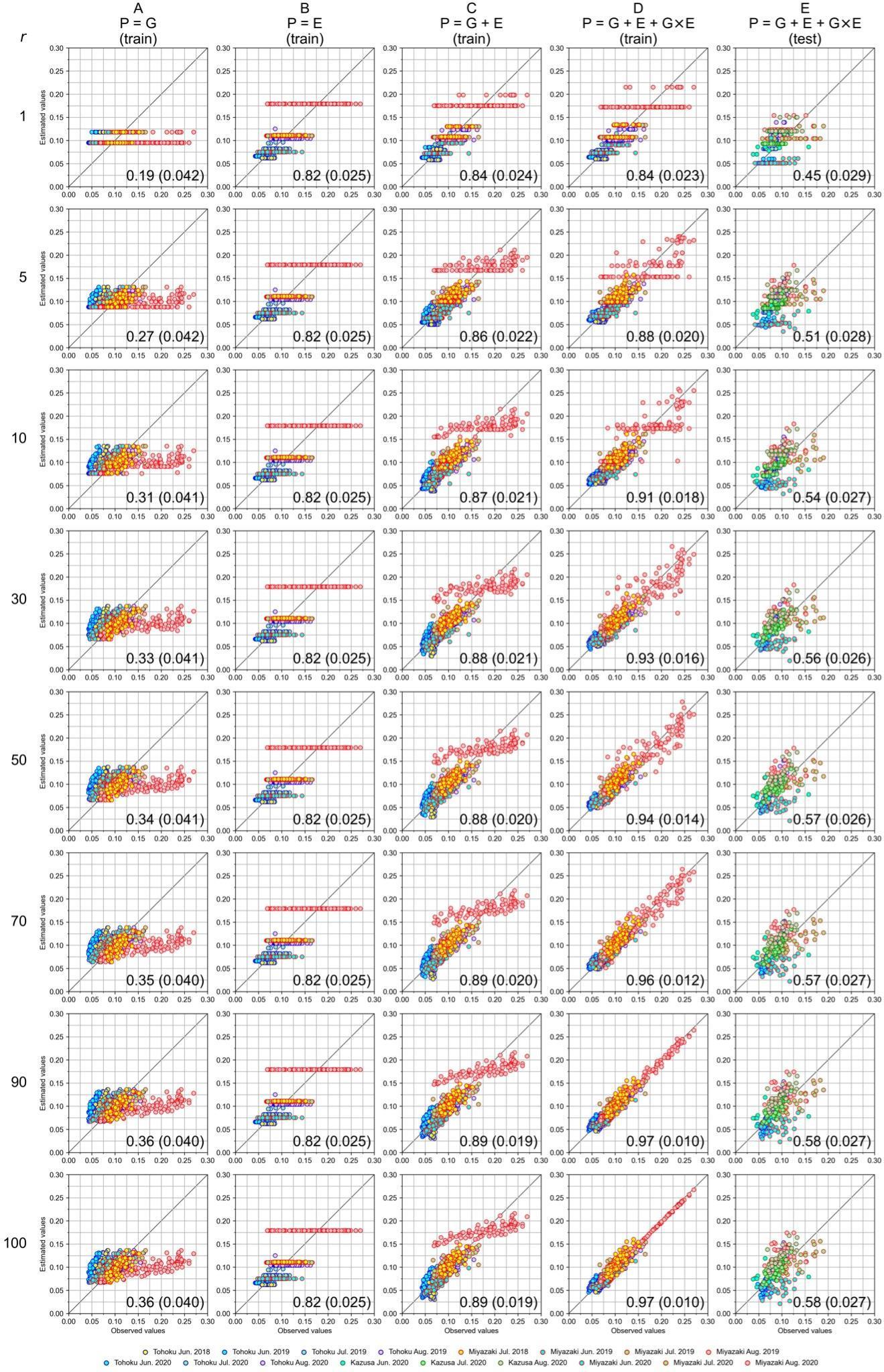


**Supplementary figure S18.** Relationship between the complexity of the models and the estimated and predicted values in relation to the *r* model. A, P = G; B, P= E; C, P = G + E; D, P = G + E + GxE; A-D are fitting results in the training data (TF and MF in 2018 and 2019); E, the prediction results in the test data (TF, KF, and MF in 2020) by the model with P = G + E + GxE generated in D; The horizontal axis shows the observed values while the vertical axis shows the estimated values (A-D) or predicted values (E). Results with the 1, 5, 10, 30, 50, 70, 90, and 100 genetic factors are compared in the vertical direction. The Pearson’s correlation coefficient between the observed values and the estimated and/or predicted values is indicated at the bottom right of each plot. The root mean squared error (RMSE) is also indicated in the parentheses.

**
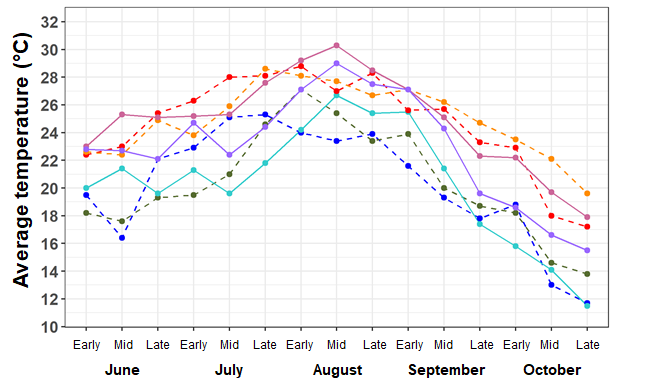
**

**Supplementary figure S19.** Ten-days average temperature (°C) in MF, TF, and KF. Red and blue colors represent MF and TF in 2018, respectively. Orange and green colors represent MF and TF in 2019, respectively. Pink, light blue, and purple represent MF, TF, and KF in 2020, respectively. Dashed line indicates the temperature from 2018-2019, solid line for the temperature in 2020.


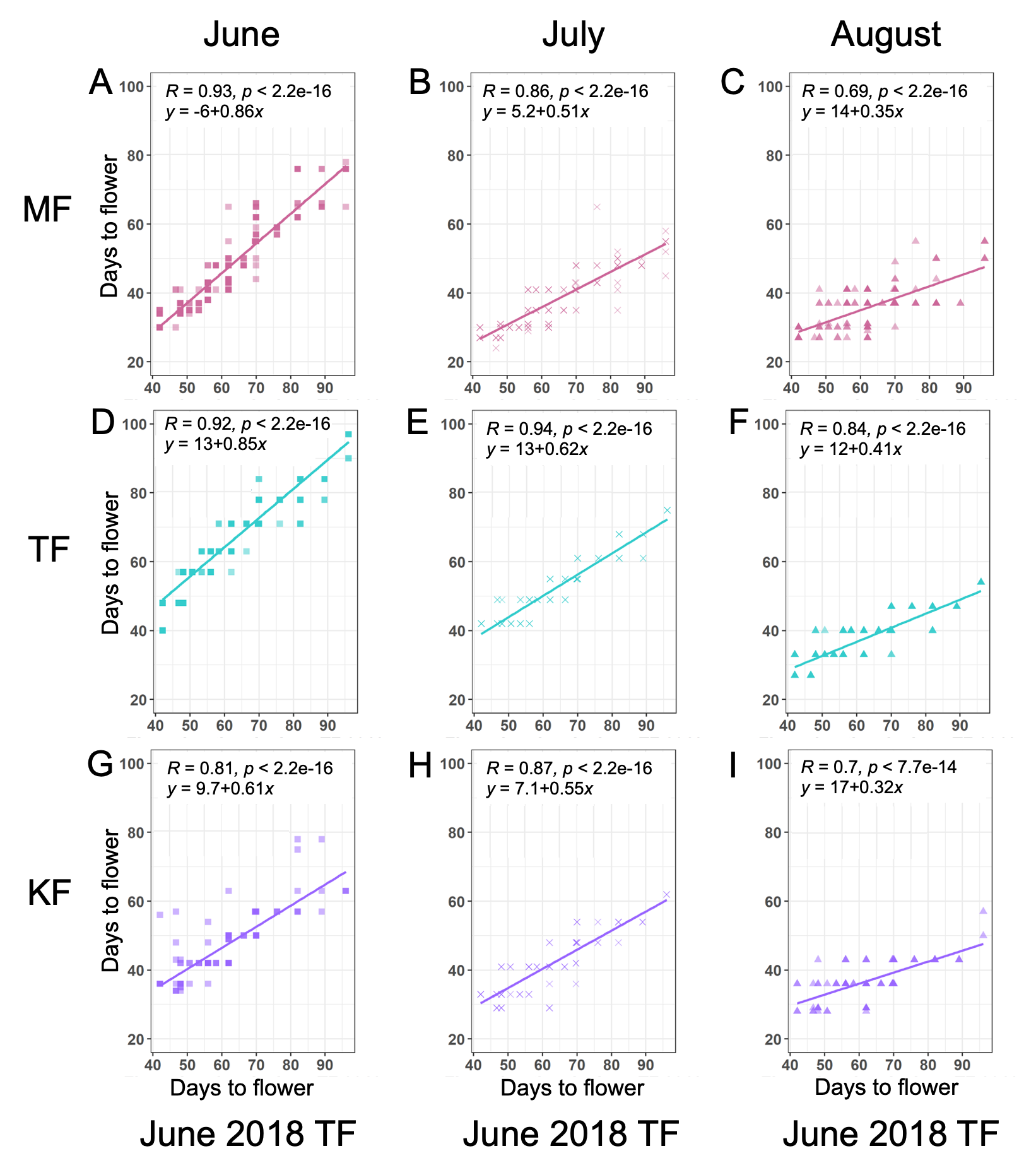


**Supplementary figure S20.** Scatter plots depicting the flowering time for 32 soybean cultivars across three different sowing times in three fields in 2020 compared with the flowering time of cultivars sown in June 2018 in TF. A, June sowing in MF; B, July sowing in MF; C, August sowing in MF; D, June sowing in TF; E, July sowing in TF; F, August sowing in TF; G, June sowing in KF; H, July sowing in KF; I, August sowing in KF. The correlation and *p* value were calculated based on Pearson’s correlation.


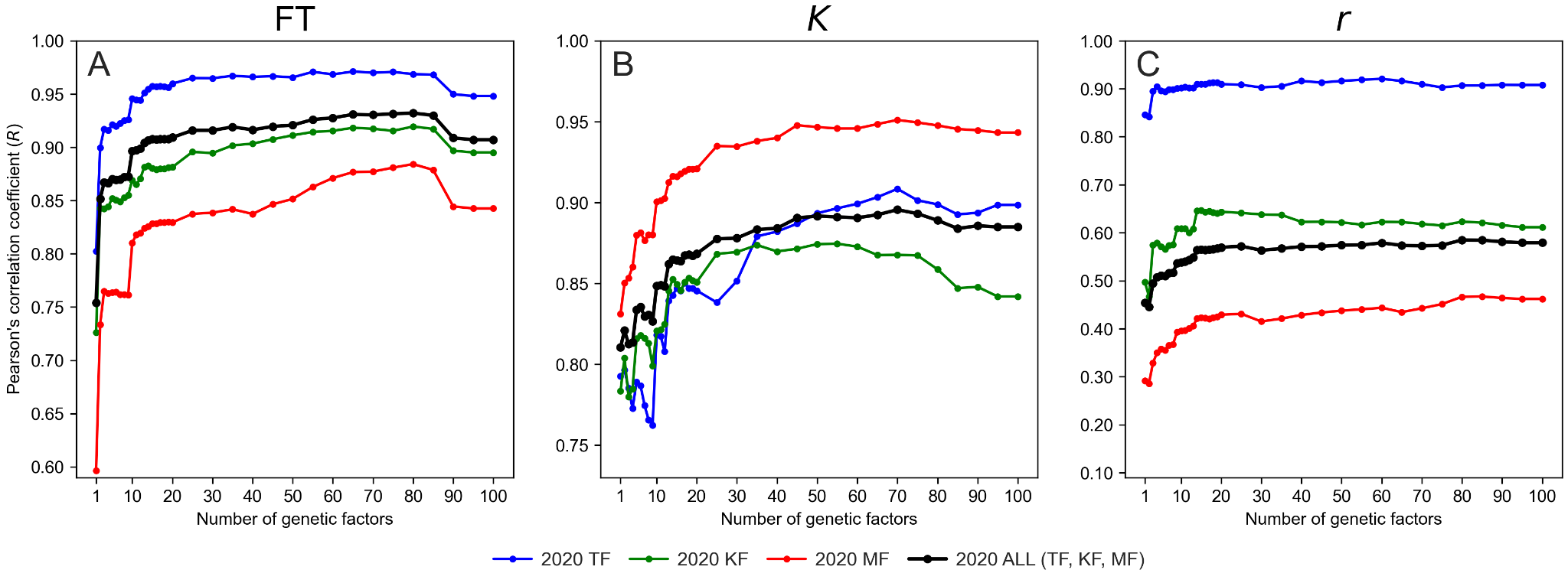


**Supplementary figure S21.** Relationship between the number of the genetic factors in the models (P = G + E + GxE) and the prediction ability in the test data in 2020. The models consisted of each number of the genetic factors (sequence variants) and the environmental factors (average temperatures, sowing date, and latitude) and their interactions. A, Relationship in relation to the FT model; B, Relationship in relation to the *K* model; C, Relationship in relation to the *r* model; The horizontal axis and the vertical axis show the number of the genetic factors and the prediction ability, respectively. The prediction ability is presented using the Pearson’s correlation coefficient. TF, KF, and MF are respectively in blue, green, and red. Result of all the three fields in total is in black.

**
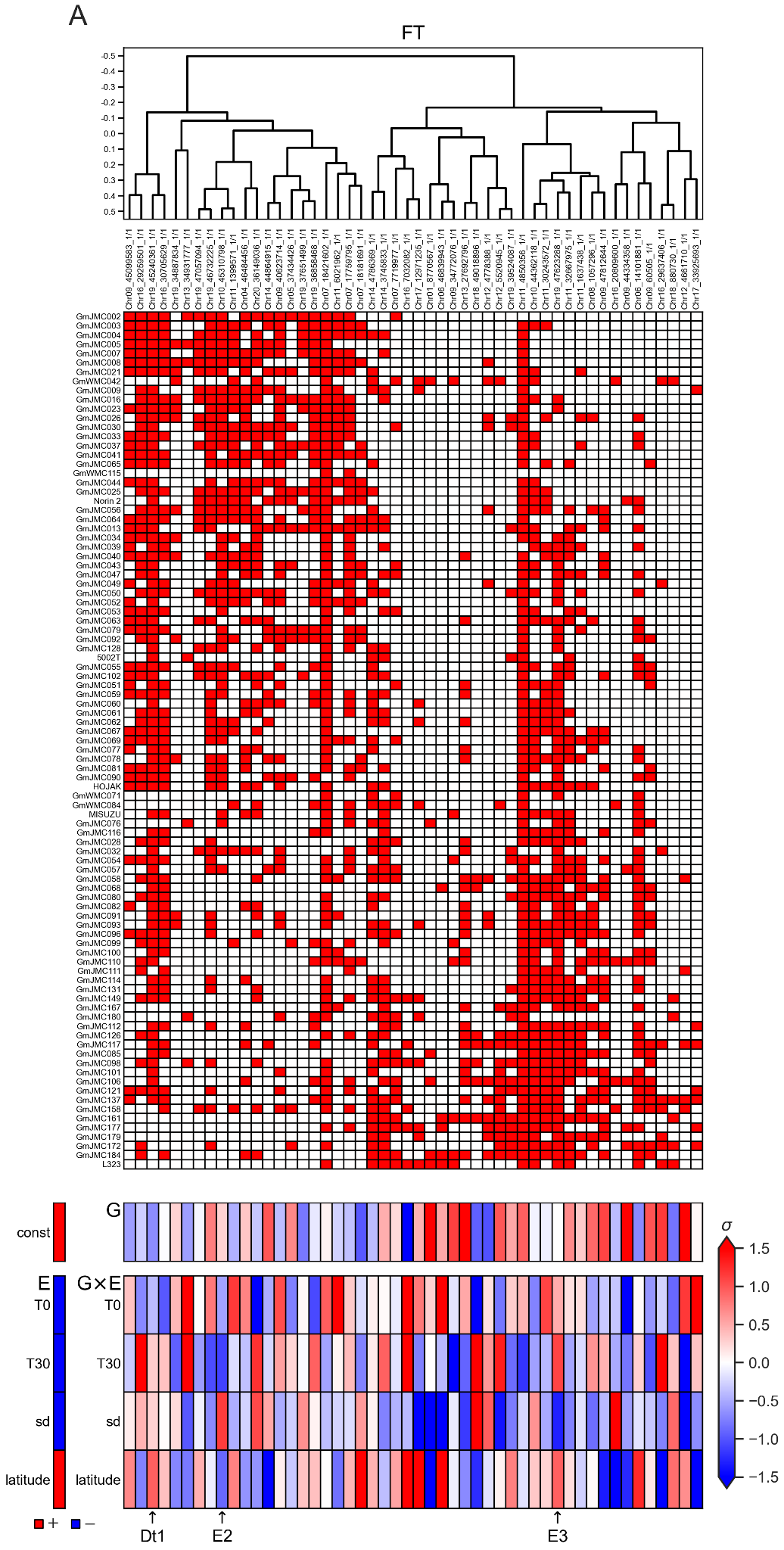

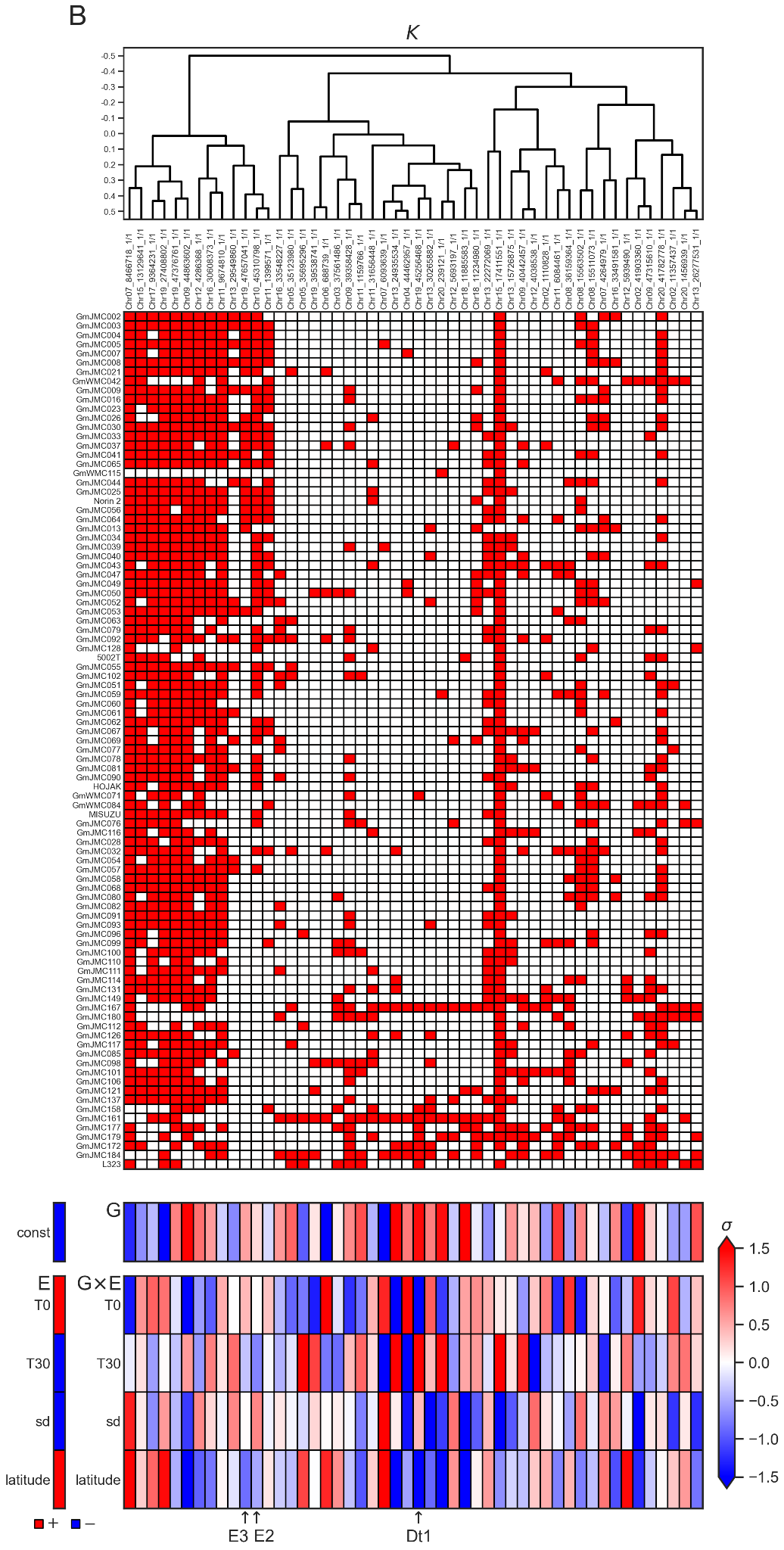

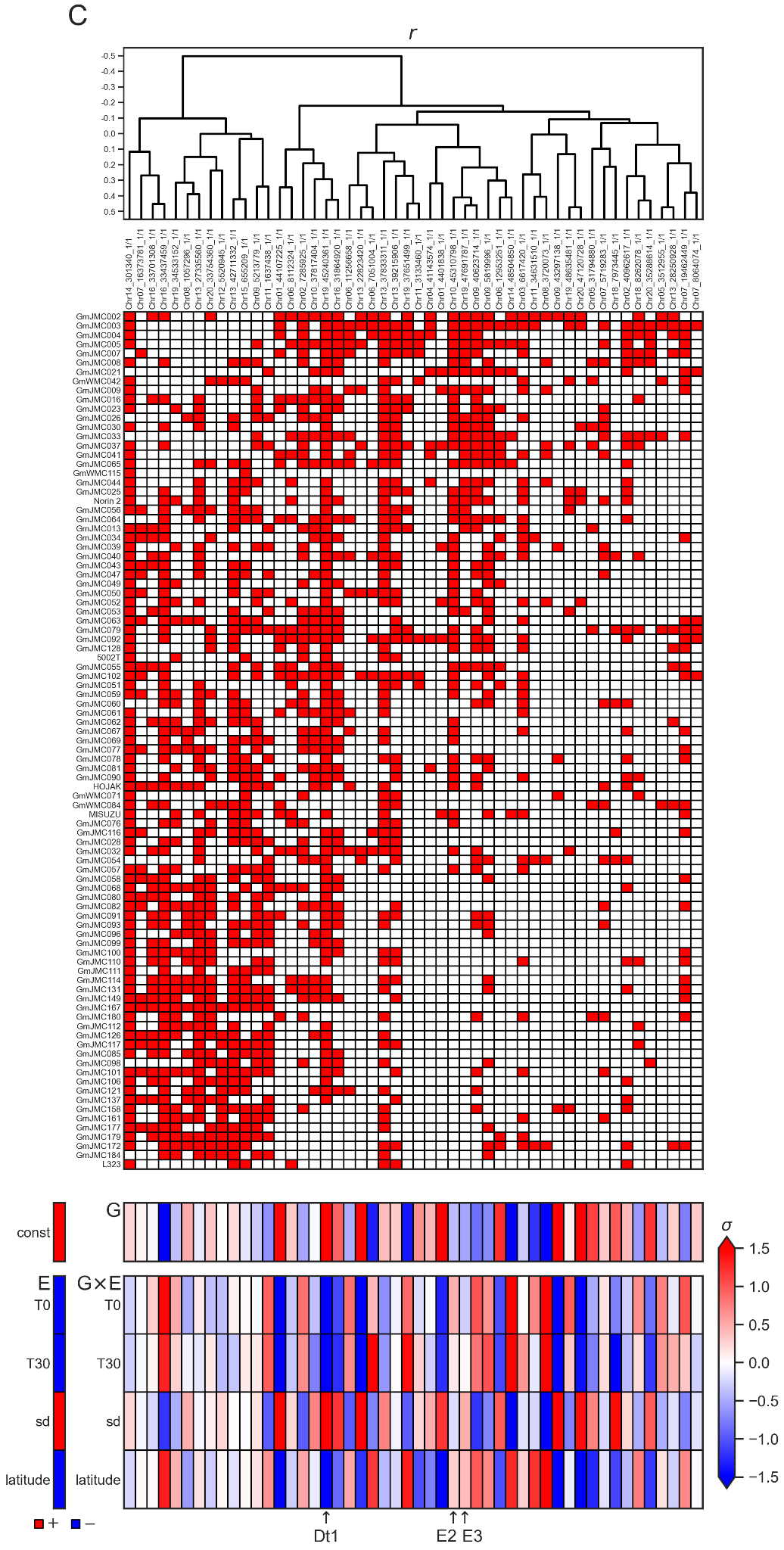
**

**Supplementary figure S22.** The distributions of the genetic factors in the 93 cultivars and their coefficients in the models. A, B, and C respectively show the 50 genetic factors selected for the FT, *K*, and *r* models (P = G + E + GxE) and their coefficients (Tables S16, S20, and S24). Each red rectangle marker in the upper part indicates that the cultivar shown on the left has the homozygous alternate allele (genetic factor) at the locus shown on the top. The 93 cultivars are arranged in the increasing order of the trait values in June 2018 in TF from the top to the bottom. The 50 loci are arranged in the order obtained by the hierarchical clustering (complete-linkage method) based on the correlation among the variant patterns in the columns. The colors (blue to red) in the lower part indicate the values of the coefficients in the model in relation to the terms used in G, E, and GxE in addition to the constant term (const). The coefficients for the constant and E are in blue or red, respectively indicating the values are less than or greater than zero. In relation to the terms G and GxE, the coefficients are indicated in blue or red in an identical manner utilizing the gradient of the colors to show the absolute value normalized by the standard deviation in the 50 loci. The contribution of a term in G and GxE to the trait value of an individual can be determined by combination of the presence/absence of the corresponding variant and the magnitude of the coefficient. The columns corresponding to *Dt1/GmTFL1*, *E2*, and *E3* are indicated by arrows.

| A | 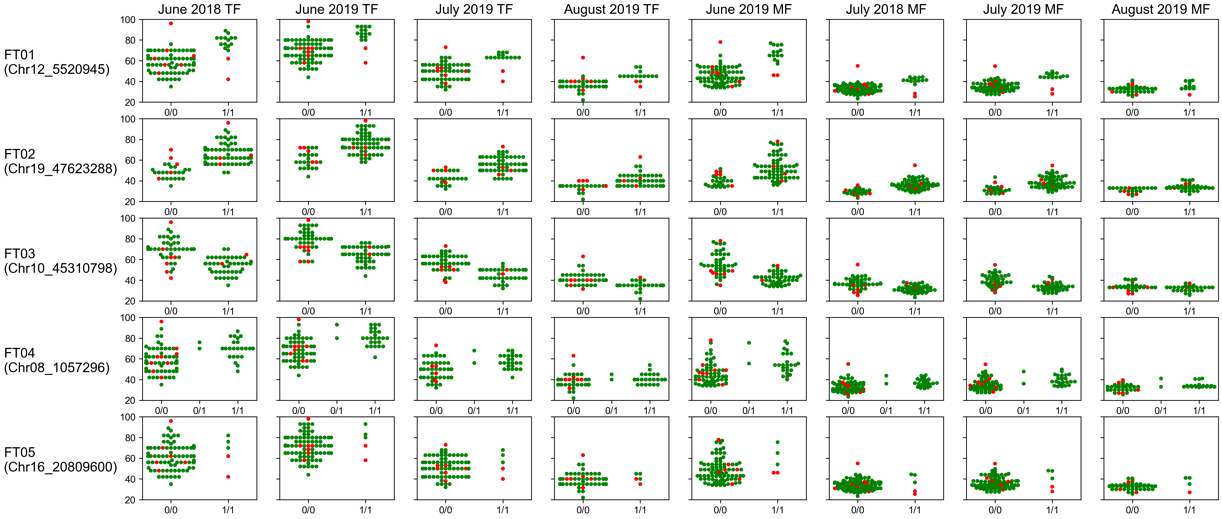 |
| --- | --- |
| B | 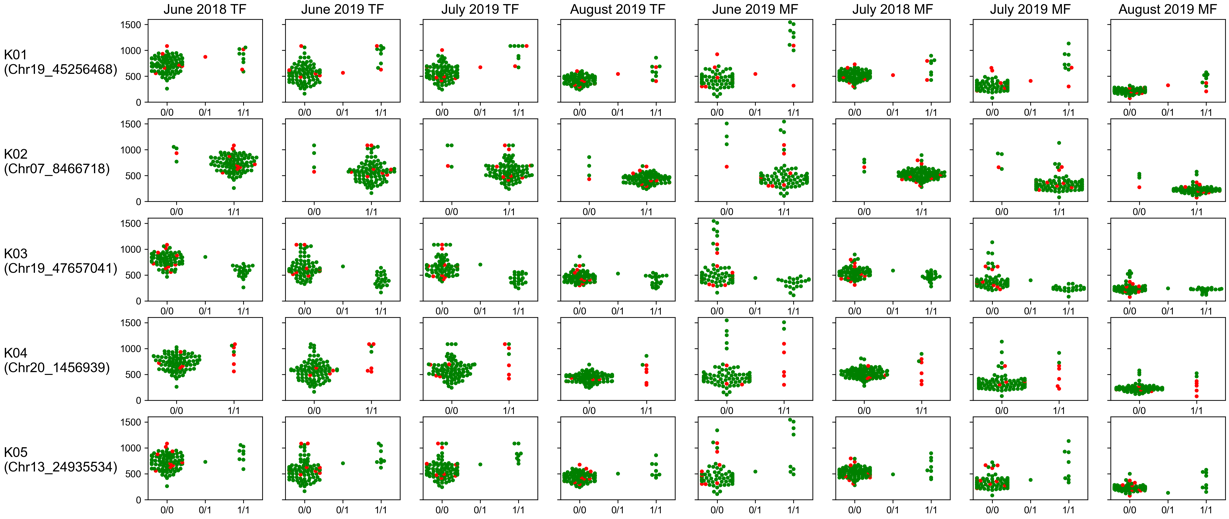 |
| C | 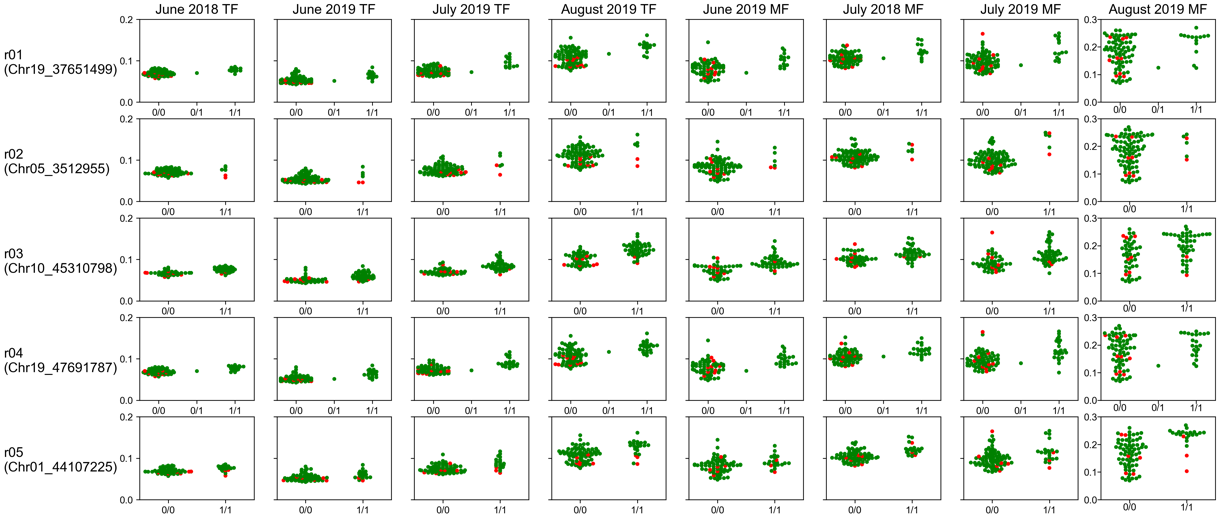 |

**Supplementary figure S23.** Relationships between the genotypes of the genetic factors and the distributions of the trait values in the eight environments in MF and TF in 2018 and 2019. A, B, and C respectively show a part of the genetic factors used in the models (FT01 to FT05, K01 to K05, and r01 to r05). Filled circles represent the cultivars and their colors (red and green) show the cluster they belong to presented in Fig. S4B. 0/0, 0/1, and 1/1 along the horizontal axis represent the genotypes of the loci (homozygous reference, heterozygous, and homozygous alternate).


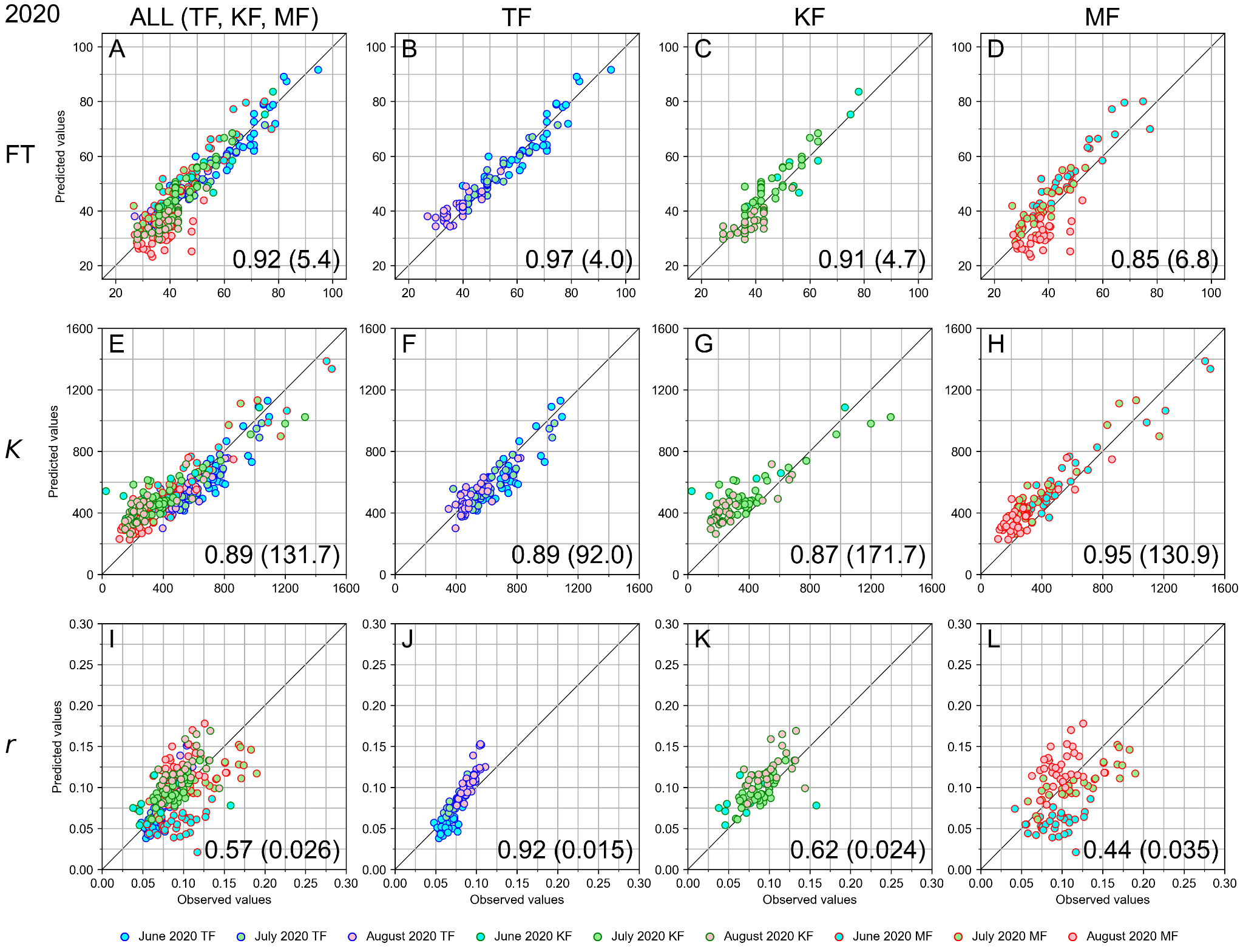


**Supplementary figure S24.** Prediction results by the models with 50 genetic factors using the test data in 2020. A, E, and I are respectively identical to Fig. 4A, 4B, and 4C. A, Results in TF, KF, and MF by the FT model; B, Results in TF by the FT model; C, Results in KF by the FT model; D, Results in MF by the FT model; E, Results in TF, KF, and MF by the *K* model; F, Results in TF by the *K* model; G, Results in KF by the *K* model; H, Results in MF by the *K* model; I, Results in TF, KF, and MF by the *r* model; J, Results in TF by the *r* model; K, Results in KF by the *r* model; L, Results in MF by the *r* model. The horizontal axis and the vertical axis show the observed values and the predicted values, respectively. Color scheme is the same as in Fig. 4. The Pearson’s correlation coefficient between the observed values and the predicted values is indicated at the bottom right of each plot. The root mean squared error (RMSE) is also indicated in the parentheses.
